# The Effect of Select SARS-CoV-2 N-Linked Glycan and Variant of Concern Spike Protein Mutations on C-Type Lectin-Receptor-Mediated Infection

**DOI:** 10.3390/v15091901

**Published:** 2023-09-09

**Authors:** Arjan Bains, Wenyan Guan, Patricia J. LiWang

**Affiliations:** 1 Chemistry and Biochemistry, University of California Merced, 5200 North Lake Rd., Merced, CA 95343, USA; abains5@ucmerced.edu; 2 Materials and Biomaterials Science and Engineering, University of California Merced, 5200 North Lake Rd., Merced, CA 95343, USA; wguan3@ucmerced.edu; 3 Molecular Cell Biology, Health Sciences Research Institute, University of California Merced, 5200 North Lake Rd., Merced, CA 95343, USA

**Keywords:** SARS-CoV-2, COVID-19, N-linked glycans, C-type lectin receptor, DC-SIGN, variants of concern, trans-infection

## Abstract

The SARS-CoV-2 virion has shown remarkable resilience, capable of mutating to escape immune detection and re-establishing infectious capabilities despite new vaccine rollouts. Therefore, there is a critical need to identify relatively immutable epitopes on the SARS-CoV-2 virion that are resistant to future mutations the virus may accumulate. While hACE2 has been identified as the receptor that mediates SARS-CoV-2 susceptibility, it is only modestly expressed in lung tissue. C-type lectin receptors like DC-SIGN can act as attachment sites to enhance SARS-CoV-2 infection of cells with moderate or low hACE2 expression. We developed an easy-to-implement assay system that allows for the testing of SARS-CoV-2 trans-infection. Using our assay, we assessed how SARS-CoV-2 Spike S1-domain glycans and spike proteins from different strains affected the ability of pseudotyped lentivirions to undergo DC-SIGN-mediated trans-infection. Through our experiments with seven glycan point mutants, two glycan cluster mutants and four strains of SARS-CoV-2 spike, we found that glycans N17 and N122 appear to have significant roles in maintaining COVID-19′s infectious capabilities. We further found that the virus cannot retain infectivity upon the loss of multiple glycosylation sites, and that Omicron BA.2 pseudovirions may have an increased ability to bind to other non-lectin receptor proteins on the surface of cells. Taken together, our work opens the door to the development of new therapeutics that can target overlooked epitopes of the SARS-CoV-2 virion to prevent C-type lectin-receptor-mediated trans-infection in lung tissue.

## 1. Introduction

Despite the rapid development and wide dissemination of SARS-CoV-2 vaccines, for the past 2 years the winter season has culminated in a surge in coronavirus infections [1]. These surges have coincided with the rise of new variants that can evade detection by the immune system: the variants of concern (VOC) B.1.1.7 (Alpha), B.1.135 (Beta), B.1.525 (Eta), B.1.617.1 (Delta) and P.1/B.1.1.28.1 (Gamma) were all identified in the late fall to winter of 2020–2021 [2,3]. B.1.1.529 (Omicron) was identified in several countries in November of 2021, and various Omicron subvariants have developed and display increased vaccine evasion. Out of these new variants, Omicron XBB.1.5 is rapidly taking over a greater proportion of COVID-19 cases globally and appears to reliably establish breakthrough infections in patients who have been vaccinated more than three times (including patients who have received bivalent boosters) [3,4,5,6].

The consistent ability of SARS-CoV-2 to avoid recognition by the immune system and establish breakthrough infections despite vaccine rollouts has led numerous members of the medical community to concede that COVID-19 is here to stay. Much like the seasonal flu, annual or even biannual booster shots are commonplace and necessary to combat the latest variants of the virus [7,8]. Even though the mutation rate of the polymerase for SARS-CoV-2 is an order of magnitude or so lower than for human immunodeficiency virus (HIV)-1, the ease of infection, relatively broad tropism and long course of infection allow for sufficient rates of mutations for the SARS-CoV-2 genome to escape immune detection and eventually establish breakthrough infection [9,10,11]. The evidence that COVID-19 will likely continue to mutate is perhaps best exemplified by how, globally, Delta strains were the dominant variant of SARS-CoV-2 for about 6 months between June and December of 2021, with nearly 100% of COVID-19 cases worldwide testing as the Delta variant for the latter 5 months of that period [6]. Yet, despite this dominance, Omicron is now the prevalent variant, with almost triple the number of spike mutations as Delta [7]. While initial selective pressure on the Omicron strain favored immune escape, it appears that Omicron strains are now undergoing mutations to increase their pathogenic activity, as shown by the increased transmissibility of Omicron BA.2 and BA.3 when compared with BA.1 and the increased fusogenic activity of more recent strains like Omicron XBB.1.5, CA.3.1 and CH.1.1 when compared with BA.2 [4,10,11,12,13,14,15]. 

The SARS-CoV-2 genome is approximately 29,900 nucleotides long and encodes sixteen nonstructural, four structural and up to six accessory proteins [16]. Since the sixteen nonstructural proteins are involved with replication and transcription, and the six accessory proteins are not incorporated into the mature virion (although they are hypothesized to impede host immune system signaling or otherwise aid in viral pathogenesis), this leaves the four structural proteins as the key factors that can affect virion architecture, determine virus infectivity, and act as antigen recognition sites for the host immune system [16,17,18]. Of these four structural proteins, one of them—the N protein—associates with the viral RNA genome on the interior of the virion [16]. The other three proteins are expressed on the surface of the viral particle and are called S (spike protein), M (membrane protein) and E (envelope protein). Perhaps the most clinically relevant protein out of the three surface proteins is the spike protein, since it directs recognition and fusion mediated via the human angiotensinogen-converting enzyme 2 (hACE2) receptor on the surface of target cells [16]. Nearly all antibodies that display neutralizing ability against SARS-CoV-2 bind to the spike protein, particularly the S1 domain [19,20,21,22]. Furthermore, RNA vaccines (Moderna mRNA-1273 and Pfizer-BioNTech BNT162b2/Comirnaty), adenoviral vaccines (AstraZeneca—Oxford ChAdOx1-S and Janssen Biologics B.V./Janssen Pharmaceutica NV COVID-19 Vaccine, Janssen) and novel mosaic/nanoparticle vaccines raise antibodies against the spike protein, further highlighting that COVID-19 breakthrough infections are largely determined by epitope modification of the spike protein [23,24,25,26,27]. Ergo, there is an urgent need to identify immutable locations (also known as “cold spots”) on the SARS-CoV-2 spike protein and use these to create variant-proof COVID-19 vaccines and prophylactics [28,29,30]. 

The S protein is a trimeric transmembrane glycoprotein comprising two domains: the S1 domain, which is responsible for attachment to target cells via the hACE2 receptor, and the S2 domain, which contains the fusion peptide and orchestrates membrane fusion and viral infection [21,31]. The S1 domain is further subdivided into an N-terminal domain (NTD), two C-terminal domains (CTD1 and CTD2) and a receptor-binding domain (RBD), which contains a surface called the receptor binding motif (RBM). The RBM is a 69-amino-acid loop that contains 17 residues that contact 20 residues in the hACE2 N-terminal helix and are integral in dictating SARS-CoV-2 tropism [31,32,33]. The spike protein can adopt two conformations: a “down” or “closed” position where the RBM is inaccessible to the hACE2 receptor and an “up” or “open” position where the RBD opens and reveals the RBM for binding to the hACE2 receptor [31,32,33]. 

**Figure 1:**
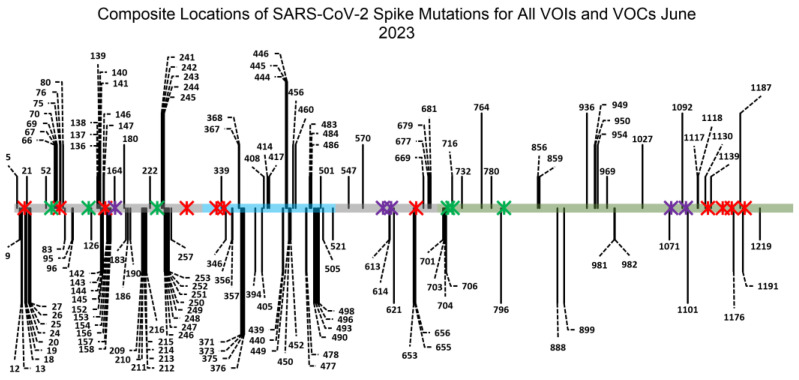
Representation of the SARS-CoV-2 spike gene amino acid sequence with all locations of VOC mutations. The S1 domain is depicted in grey (residues 14 to 685), which is further subdivided into four domains: the N-terminal domain (NTD, residues 14 to 306), the receptor-binding domain (RBD, residues 319 to 527, depicted in teal) and the two C-terminal domains, C-terminal domain 1 (CTD1, residues 527 to 591) and C-terminal domain 2 (CTD2, residues 591 to 686). Within the receptor-binding domain is the receptor-binding motif (RBM, residues 438 to 506), which encompasses the key amino acids that mediate SARS-CoV-2 spike protein binding to the host hACE2 receptor. The S2 domain is depicted in grey-green and ranges from residues 686 to 1273. Black lines indicate the locations of point mutations across VOCs (for a list of the specific VOCs summarized here, see Figure S2). Red, green and purple Ж crosses depict the locations of N-linked glycans. Red crosses indicate complex glycans, green crosses indicate high-mannose glycans and purple crosses depict hybrid glycans and glycans that were inconsistently identified as being complex or high-mannose, based on an analysis of existing literature (Figures S3 and S4). While mutations have occurred in VOCs immediately adjacent to glycan sites, there have been no variants that have mutations at the asparagine residues where N-linked glycosylation is present [41,55,56].

The SARS-CoV-2 S protein also has 22 N-linked glycosylation sites and 2 O-linked glycosylation sites [34,35,36,37]. Glycans on viral spike proteins perform several functions, including being recognized by receptors on host cells, maintaining protein stability and shielding viral spike epitopes from the host immune system [34,38,39,40]. In the case of SARS-CoV-2, it has been shown that lectin receptors such as DC-SIGN, L-SIGN, CLEC4G and SIGLEC can bind to the glycans on the surface of the S protein and “hold” the virus in close proximity to cells, thereby facilitating more efficient infection [41,42,43,44,45]. There is also evidence that soluble lectin proteins—such as galectins—can bind to virions and bring them in close proximity to host cells, also facilitating infection [46,47,48]. This is particularly pertinent for the progression of COVID-19 since SARS-CoV-2 virions are transmitted via airborne routes and infection initiates in the proximal airways [42,49]. While lung tissue acts as the point of entry for SARS-CoV-2 virions, the respiratory system only modestly express hACE2. It has been shown that lectin-receptor-mediated trans-infection—whereby a cell type that expresses a lectin receptor facilitates the transfer and entry of SARS-CoV-2 virions to cells that mildly express hACE2—is the primary process by which COVID-19 initiates infection in the lungs [42,45,50,51,52]. This is further supported by the fact that lung tissue is highly patrolled by the innate immune system, with DC-SIGN+ dendritic cells being found throughout the lung interstitium and in the airways (Figure S1) [43]. Most significantly, in contrast to other viruses like HIV and Ebola virus, almost none of the SARS-CoV-2 variants of concern (VOC) have displayed any alteration at the key asparagines where N-linked glycosylation is found on the SARS-CoV-2 spike [41,53,54,55,56]. Based on an analysis of a database of high-quality SARS-CoV-2 genomes from human patients, none of the asparagines where N-linked glycosylation was present on the SARS-CoV-2 spike showed a mutation rate higher than 0.3%, and none of the S1-domain glycans showed a mutation rate higher than 0.06% (Table 1) [57]. Considering the number of mutation sites across all SARS-CoV-2 VOCs (Figure 1)—109 mutations in the 1273 aa long spike—and the fact that mutations are detectable in patients as early as 4 days into COVID-19 infection, the immutability of the SARS-CoV-2 spike glycans is remarkable [58].

In the following work, key glycans that are necessary for maintaining the functionality of the SARS-CoV-2 spike in facilitating both hACE2-mediated direct infection and DC-SIGN-mediated trans-infection were identified. If specific glycan mutations diminished the infectivity of the virus, then there is likely a strong selective pressure for those glycans to be preserved no matter what possible mutations may occur in future variants of SARS-CoV-2. This would provide key epitope locations that could act as “cold spots” for the development of a variant-proof vaccine or prophylactic [42,53]. Particular focus was given to those spike glycans that were in close proximity to the RBD, since it was hypothesized that impairing the conformational dynamics of the receptor-binding domain would have the highest likelihood of preventing infection. Indeed, this is likely why numerous vaccines currently in development make use of the RBD as the antigen rather than the full-length spike [10,19,25,59]. However, since the eventual goal of this work is to identify SARS-CoV-2 spike protein epitopes beyond what current therapeutics target, N-linked glycans that were proximal to the spike RBD—but not directly on it—were selected. In this study, the glycans at N17, N61, N74, N122, N149, N165 and N234 were assessed.

In addition to evaluating the importance of S1-domain glycans, we also assessed how spike glycoproteins from certain VOC strains affected the infectivity of SARS-CoV-2 pseudotyped virions. The impact of how spike proteins from some COVID-19 strains alter direct infectivity has already been tested in vitro, but, to our knowledge, no in vitro lectin-receptor-mediated trans-infection tests have been performed to compare the susceptibility of different strains of SARS-CoV-2 [4,10,11,14,15,60,61,62]. We report the differences in DC-SIGN-mediated trans-infection for Wuhan CoV-19, D614G, Delta and Omicron BA.2 pseudovirions on our assay system.

## 2. Materials and Methods

### 2.1. Cell Lines 

All cell lines were maintained in Dulbecco’s Modified Eagle Medium (DMEM) (ThermoFisher Cat # 11965-092, Waltham, MA, USA) with specific additives for each cell type. These cells include:

- HEK-293FT (Homo sapiens embryonic kidney cells; a generous gift from Dr. David Gravano, University of California, Merced).
- HEK-293T hACE2+ cells (Homo sapiens embryonic kidney cells; obtained through BEI Resources, NIAID, NIH: Human Embryonic Kidney Cells [HEK-293T] Expressing Human Angiotensin-Converting Enzyme 2, HEK-293T-hACE2 Cell Line, NR-52511).
- 3t3 wild-type cells (Mus musculus mouse embryonic fibroblasts; obtained through the NIH HIV Reagent Program, Division of AIDS, NIAID, NIH: NIH-3T3 Cells, ARP-9946; contributed by Drs. Thomas D. Martin and Vineet N. KewalRamani).
- 3t3 DC-SIGN+ cells (Mus musculus mouse embryonic fibroblasts; obtained through the NIH HIV Reagent Program, Division of AIDS, NIAID, NIH: NIH 3T3 DC-SIGN+ Cells, ARP-9947; contributed by Drs. Thomas D. Martin and Vineet N. KewalRamani).

Both HEK-293FT cells and HEK-293T hACE2+ cells were maintained in T-75 flasks (Stellar Scientific Cat # SKU:TC30-120, Baltimore, MD, USA) within a 37 °C humidified incubator at 4.5% CO_2_. HEK-293FT cells and HEK-293T hACE2+ cells were cultured in DMEM supplemented with 25 mM HEPES (ThermoFisher Cat # 11344041, Waltham, MA, USA), 2 mM L-glutamine (R&D Systems Cat # R90010, Minneapolis, MN, USA), 250 μg mL^−1^ G418 sulfate (Corning Life Sciences Cat # 30-234-CI, Tewksbury, MA, USA) and 10% fetal bovine serum (R&D Systems Cat # S11150, Minneapolis, MN, USA). This medium is henceforth referred to as 293 Medium. 

3t3 cells were chosen because they have been demonstrated to be deficient in a variety of lectin receptors, including DC-SIGN (CD209), LOX1 (OLR1) and CLEC family proteins, according to a gene expression database (NIH3t3 cells, harmonizome.com). Both 3t3 wild-type cells and 3t3 DC-SIGN+ cells were maintained in T-75 flasks within a 37 °C humidified incubator at 4.5% CO_2_. 3t3 wild-type cells and 3t3 DC-SIGN+ cells were cultured in DMEM supplemented with 2 mM GlutaMAX (ThermoFisher Cat # 35050-061, Waltham, MA, USA), 100 U mL^−1^ of penicillin–streptomycin solution (Cytiva HyClone Cat # SV30010, Marlborough, MA, USA) and 10% fetal bovine serum (R&D Systems Cat # S11150, Minneapolis, MN, USA). This medium is henceforth referred to as 3t3 Medium.

When a flask reached 80–95% confluency, cells were passaged 1:10 with 0.05% trypsin–EDTA (ThermoFisher Cat # 25300-062, Waltham, MA, USA), used to detach cells from the surface of plastic flasks. Trypsin was not permitted to exceed a contact time of 5 min for any cell line. 

### 2.2. Pseudovirus Production

Pseudovirus plasmids were obtained through BEI Resources, NIAID, NIH. SARS-CoV-2 spike plasmids were originally purchased from Addgene. SARS-CoV-2 spike pseudotyped HIV virions were produced using a protocol inspired by Crawford et al., 2020 [63]. Briefly, HEK-293FT cells were maintained in 293 Medium and passaged once cells reached 70–100% confluency. The day prior to transfection, HEK-293FT cells were seeded at a density of approximately 2.5 × 10^6^ cells in 8–10 mL of 293 Medium in a 100 mm tissue culture petri dish or approximately 0.2 × 10^6^ cells in 2–3 mL of 293 Medium in a 6-well tissue culture plate (batches of virus were made in either of the two plate types). Cells were allowed to recover for 12–16 h in a humidified incubator at 37 °C and 5% CO_2_. 

Petri dishes/6-well plates were then withdrawn from incubators and cells were assessed for confluency and adhesion. If the cells in petri dishes were 70% confluent and adherent, the medium was gently replaced with 8 mL of fresh 293 Medium. If the cells in 6-well plates were 70% confluent and adherent, the medium was gently replaced with 3 mL of fresh 293 Medium. The petri dish/6-well plate was returned to the incubator for approximately 45 min while the plasmids and transfection reagent were prepared. For a 100 mm petri dish, the following plasmids were mixed in 900 μL of serum-free commercial DMEM in a sterile 1.5 mL centrifuge tube: 5 μg of lentiviral backbone Luciferase-IRES-ZsGreen (BEI Resources NR-52516, Manassas, VA, USA) vector, 1.1 μg each of vectors HDM-Hgpm2 (BEI Resources NR-52517, Manassas, VA, USA), pRC-CMV-Rev1b (BEI Resources NR-52519, Manassas, VA, USA) and HDM-tat1b (BEI Resources NR-52518, Manassas, VA, USA) and 1.7 μg of vector pCMV14-3X-Flag-SARS-CoV-2 S (Addgene Cat # 145780, Watertown, MA, USA). For a 6-well tissue culture plate, the same plasmids were mixed in 185 μL of serum-free commercial DMEM in a sterile 1.5 mL centrifuge tube: 1 μg of lentiviral backbone Luciferase-IRES-ZsGreen (BEI Resources NR-52516, Manassas, VA, USA) vector, 0.22 μg each of vectors HDM-Hgpm2 (BEI Resources NR-52517, Manassas, VA, USA), pRC-CMV-Rev1b (BEI Resources NR-52519, Manassas, VA, USA) and HDM-tat1b (BEI Resources NR-52518, Manassas, VA, USA) and 0.34 μg of spike vector pCMV14-3X-Flag-SARS-CoV-2 S (Addgene Cat # 145780).

For the creation of pseudovirus with N-linked glycan spike mutations, we either performed site-directed mutagenesis using PCR or ordered segments of DNA with specified glycan mutations flanked with HindIII and AvrII cut sites (Twist Bioscience, South San Francisco, CA, USA). Segments of DNA were ligated into spike vector pCMV14-3X-Flag-SARS-CoV-2 S, which had been digested with HindIII-HF (New England Biolabs Cat # R3104S, Ipswich, MA, USA) and AvrII (New England Biolabs Cat # R0174S, Ipswich, MA, USA). To verify the presence of asparagine-to-glutamine spike mutations, the vectors were sequenced (UC Berkeley DNA Sequencing Facility, Berkeley, CA, USA). These mutated pCMV14-3X-Flag-SARS-CoV-2 S spike vectors were used in the transfection mixture as described above.

For the creation of pseudovirus with a spike protein with the D614G mutation, PCR was performed as described for the N-linked glycan spike mutations and then used in the same transfection mixture specified above. For the creation of Delta pseudovirus, transfection was performed as described above, except that pcDNA3.3-SARS2-B.1.617.2 (Addgene Cat # 172320) was used as the spike protein vector in lieu of pCMV14-3X-Flag-SARS-CoV-2 S. For the creation of Omicron BA.2 pseudovirus, transfection was performed as described above except that pcDNA3.3_SARS2_omicron BA.2 (Addgene Cat # 183700) was used as the spike vector for transfection in lieu of pCMV14-3X-Flag-SARS-CoV-2 S.

After adding all plasmids, the solution was mixed by pipetting up and down approximately 10 times. For the 100 mm petri dish samples, 30 μL of XtremeGENE HP Version 9 (Roche Cat # 06366546001, Mannheim, Germany) was added directly to the solution. For the 6-well-tissue-culture-plate samples, 6 μL of XtremeGENE HP Version 9 was added directly to the solution. The viruses with additional structural proteins were exclusively made in 100 mm petri dishes, but instead of using 30 μL of XtremeGENE HP Version 9, 42 μL of XtremeGENE Version 9 was added to the solution to accommodate the increased volume from adding the structural protein vectors. The contents of the tube were again mixed by pipetting up and down approximately 10 times before being allowed to incubate at room temperature for 20–25 min. The petri dish/6-well plate was then retrieved from the 37 °C incubator, and the DNA + XtremeGENE HP mixture was dripped over the HEK-293FT cells. The transfected HEK-293FT cells were then returned to the incubator and allowed to recover for 12–18 h overnight. After 12–18 h, petri dishes/6-well plates were again removed from the incubator and the cell media were gently replaced with fresh, prewarmed 293 Medium (10 mL for 100 mm petri dishes, 4 mL for 6-well plates). Transfected cells were returned to the incubator. After an additional 48 h (60–66 h total post-transfection), petri dishes were removed from the incubator and the medium was gently removed and transferred to a 15 mL tube. This 15 mL tube was then briefly centrifuged at 750 RPM (105 RCF) for 3 min to pellet any large cell clumps. The supernatant, now containing pseudotyped lentivirus, was filtered through a 0.45 µm syringe filter and stored as 450 μL aliquots in low-binding 1.5 mL tubes (ThermoFisher Cat # 90410, Waltham, MA, USA). Aliquots were stored at −75 °C until use in future assays.

### 2.3. Pseudovirus Titration 

Each SARS-CoV-2 spike mutant was produced at least twice, yielding at least two batches of pseudovirus for each spike variant. Every batch was titrated using an XpressBio p24 ELISA kit plate according to the manufacturer’s instructions (XpressBio Cat # XB-1000, Frederick, MD, USA). The levels of p24 in each well were measured on a ClarioStar Plus microplate reader set to 450 nm (BMG Labtech, Ortenberg, Germany).

### 2.4. Virus Infectivity Assays

Each well of a clear 96-well cell-culture plate was coated with 25 μL of 0.1 mg/mL poly-L-lysine (ScienCell Research Laboratories, Cat # 0413, Carlsbad, CA, USA). The plate was returned to the 37 °C incubator for 1 to 36 h. After this time, the 96-well plate was removed from the incubator and the poly-L-lysine solution was pipetted out. Wells were then rinsed twice with 40 μL of sterile, ultrapure deionized water before being set aside in preparation for seeding. 

For trans-infection experimental samples, 5000–15,000 3t3 DC-SIGN+ cells were seeded in triplicate into the wells of a pre-prepared poly-L-lysine-conditioned 96-well plate as described above. For trans-infection negative control samples, 3t3 wild-type cells were seeded instead. The 96-well plate was returned to the 37 °C humidified incubator at 4.5% CO_2_ in order to allow the 3t3 cells to adhere and recover for 8–12 h. After recovery, the 96-well plate was retrieved from the incubator and the medium was gently pipetted out of the wells. Within 1 min of pipetting out the medium, 15 μL of fresh 3t3 Medium was added to the wells to prevent the cells from desiccating. Then, 25 μL of the requisite SARS-CoV-2 pseudovirus variant was added to the wells. For negative control samples and direct-infection samples, 25 μL of the requisite SARS-CoV-2 pseudovirus variant was dispensed into empty wells that had been pre-treated with poly-L-lysine. The 96-well plate was returned to the 37 °C humidified incubator at 4.5% CO_2_ for 2–4 h to allow virion capture. 

After 2–4 h had elapsed, the 96-well plate was retrieved from the incubator and the experimental wells were rinsed twice with 40 μL of fresh 3t3 Medium. To do so, the virus medium was gently pipetted out of the wells. Within 1 min of pipetting out the medium, 40 μL of fresh 3t3 Medium was dispensed into each well to prevent desiccation. Wells were allowed to sit for 1 min, then the medium was pipetted out of the wells to rinse out unbound virions. A total of 40 μL of fresh 3t3 Medium was once again dispensed into each well, incubated for 1 min, and once again pipetted out. At this point, 15,000–25,000 HEK-293T hACE2+ cells in 293 Medium were dispensed into each well. As mentioned above, HEK-293T hACE2+ cells were added to wells within 1 min of medium removal to avoid desiccation of 3t3 cells and captured virions. For direct-infection samples, no rinsing was performed. Instead, HEK-293T hACE2+ cells were dispensed directly into the 25 μL of SARS-CoV-2 pseudovirus variant in each well. After HEK-293T hACE2+ cells were added to wells, the 96-well plate was returned to the incubator.

At a time 12–16 h later, the 96-well plate was retrieved from the humidified incubator and 150 μL of fresh, pre-warmed 293 Medium was added over the top of each well to ensure that cells remained alive and viable for the duration of the experiment. The 96-well plate was returned to the 37 °C incubator for an additional 36 to 48 h. After 36–48 h, the 96-well plate was retrieved from the humidified incubator. Bright-Glo Luciferase Reagent (Promega Corp., Cat # E2610, Madison, WI, USA) was thawed out and protected from light until use. A total of 160–180 μL of the medium in each of the infectivity plate wells was pipetted out, leaving about 30 μL of the medium in each well after accounting for evaporation during the course of the experiment. A total of 30 μL of Bright-Glo reagent was added over the top of the wells and given 2–4 min to lyse cells. The contents of each well were then transferred to a white-backed 96-well plate and the luciferase signal was read on a CLARIOstar Plus microplate reader (BMG Labtech, Cary, NC, USA) with a 3600 gain and a 1 s normalization time. All samples were run in triplicate with at least two separate biological replicates for each condition. Furthermore, at least two different pseudoviral batches of each SARS-CoV-2 spike mutation were tested.

For mannan inhibition of DC-SIGN-mediated trans-infection, mannan polymers from Saccharomyces Cerevisiae were utilized (Sigma-Aldrich Cat # M7504-100MG, Saint Louis, MO, USA). A quantity of 100 mg of mannan was dissolved in 40 mL of ultrapure water to create a 2.5 mg/mL stock solution of mannan. When performing a trans-assay, 64 μL of the 2.5 mg/mL mannan stock was diluted to a final volume of 3 mL by adding 2.936 mL of 3t3 Medium to obtain a final concentration of 0.053 mg/mL (53.3 μg/mL) for the solution of mannan in 3t3 Medium. A total of 15 μL of this 0.053 mg/mL mannan solution was used instead of 15 μL of standard 3t3 Medium to prevent cells from desiccating just prior to adding 25 μL of pseudovirus 3t3 cells. Upon the addition of pseudovirus, the final mannan concentration in wells was at 0.02 mg/mL (20 μg/mL). 

### 2.5. Pseudovirus Precipitation and Deglycosylation 

To deglycosylate the SARS-CoV-2 pseudovirus samples, aliquots of virus were removed from −75 °C storage and allowed to thaw at room temperature. Once aliquots had fully thawed, but had not yet warmed to ambient temperature, samples were taken into biosafety cabinets and requisite volumes of cold Lentivirus Precipitation Solution (ALSTEM Cell Advancements Cat # VC100, Richmond, CA, USA) were added to the tubes according to the manufacturer’s protocol with some modifications. Briefly, one volume of cold Lentivirus Precipitation Solution was added to four volumes of virus solution (typically, 100 μL of Lentivirus Precipitation Solution into 400 μL of virus aliquot). Samples were mixed by gently pipetting up and down for 30 s, and then tubes were capped and mixed by inverting for another 60 s. Samples of lentivirus were then placed into a 4 °C refrigerator to incubate for 3 to 5 h. Lentivirus aliquots were then retrieved from the refrigerator and centrifuged in a 4 °C refrigerated centrifuge at 2500 rcf for 30 min. Samples were inspected for 10 s and then centrifuged for an additional 5 min at 10,000 rcf. Aliquots were returned to the biosafety cabinet, and most of the supernatant was gently removed (for a 400 μL aliquot with 100 μL of Lentivirus Precipitation Solution, approximately 450 μL of supernatant was removed at this stage). Then, aliquots were centrifuged for 5 min at 10,000 rcf in a 4 °C refrigerated centrifuge. Samples were again retrieved and returned to a biosafety cabinet. The remaining supernatant was gently pipetted out of the tube, with care to not disturb any potentially pelleted viruses that were at the bottom of the tube (the pelleted virus is not always visible). 

Within 2 min of removing the remaining supernatant, 10 μL of cold, ultrafiltered, autoclaved, deionized water was added to the virus pellets and mixed gently by pipetting about 10 times. Taking into account leftover medium supernatant in the virus aliquot tube, lentivirus pellets were resuspended in approximately 15 μL of liquid at this time. A total of 5 μL of cold, sterile PBS was added to the sample, then an additional 20 μL of cold, ultrafiltered, autoclaved, deionized water was added to take the virus solution to a final volume of approximately 40 μL. 

For deglycosylation, 4.5 μL of GlycoBuffer 2 (10X) (New England Biolabs Component # B3704SVIAL, Ipswich, MA, USA) was added to the resuspended virus aliquot. Then, 4.0 μL of PNGase F (New England Biolabs Component # P0704SVIAL, Ipswich, MA, USA) was added to the virus. Note that both of these components are packaged in New England Biolabs Catalog # P0704S, Ipswich, MA, USA. To create control non-deglycosylated virus, 4.0 μL of PNGase F was aliquoted into a low-binding 1.5 mL tube and heat-inactivated at 80–90 °C for 10 min. 

Once PNGase F was added to the resuspended virus aliquot, samples were placed into a 37 °C incubator for 4 to 7 h to allow deglycosylation of the samples. After the requisite time had passed for deglycosylation, virus samples were retrieved from the 37 °C incubator and virus samples were diluted back to their initial volume using fresh, cold 3t3 Medium (for example, a viral aliquot that was 400 μL when it was initially thawed, then pelleted and taken to a 45–50 μL deglycosylase reaction volume, was taken back to a final volume of 400 μL using approximately 350 μL of cold 3t3 Medium). Then, deglycosylated virus was used in virus infectivity assays as described in Section 2.4, above. 

## 3. Results

### 3.1. Some, but Not All, SARS-CoV-2 Spike NTD Glycans Affect hACE2-Mediated Infectivity

**Figure 2:**
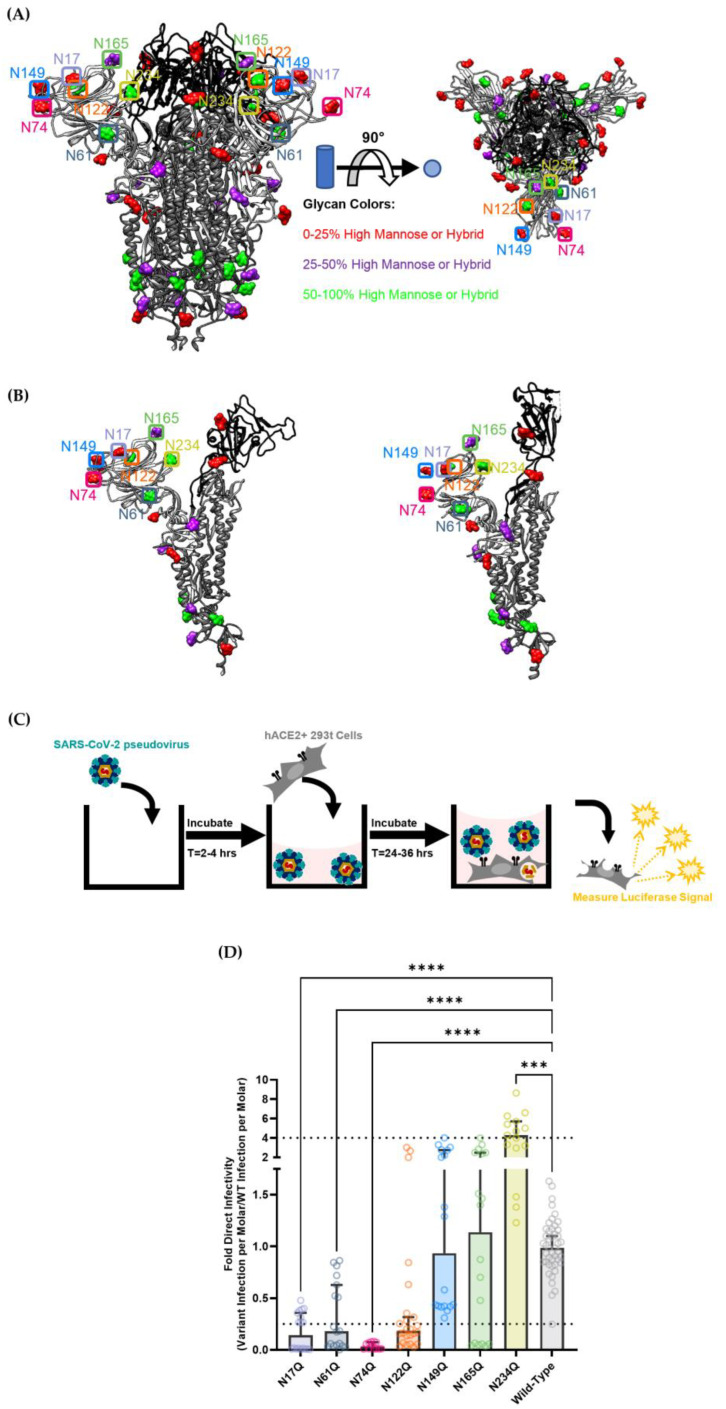
(**A**) Depiction of the trimeric SARS-CoV-2 spike with glycans. The amino acid backbone is represented as a ribbon structure: The S2 domain is dark gray, the S1 receptor-binding domain is black, and the remainder of the S1 domain outside of the RBD is light grey. The locations of N-linked glycans are depicted as space-filling models of a representative monosaccharide. High-mannose glycans are depicted in green. Complex glycans are depicted in red. Hybrid glycans or glycans that have less than a 50% consensus between high-mannose and complex type based on existing literature are depicted in purple. The left side of the picture is a side profile of the trimeric spike protein. The right side is a top view of the trimeric spike protein. The RBD closed conformation structures are adapted from PBD ID: 7NT9 by Rosa, A. et al. (**B**) Depiction of the SARS-CoV-2 spike monomer. The RBD down/closed conformation is on the left while the RBD up/open conformation is on the right. Glycans are depicted and color coded as in 2A. The RBD closed conformation structures are all from PBD ID: 7NT9 by Rosa, A. The RBD up subunit is from PDB ID: 6VYB by Walls, A.C. et al. (**C**) Simplified depiction of the direct infection process utilized for experiments. Viral samples were incubated in wells for 2–4 h before adding cells to viral suspensions. This was done to more closely mimic the incubation step of the trans-infection procedure (Figure 3). (**D**) Direct infectivity of selected SARS-CoV-2 spike S1-domain N-linked glycosylation mutants. Each pseudoviral strain was titered using a p24 ELISA kit, then the infection signal for each virus was divided by the molarity of the virus sample to obtain each variant’s infectivity per mole. Infection per mole for each mutant strain was then divided by the infection per mole of the wild-type nCov-19 Wuhan-strain SARS-CoV-2 spike pseudovirus to obtain the fold direct infectivity. Dashed lines depict a four-fold difference in viral infectivity (threshold of significance based off of Li, Q. et al.) [65]. Graphs depict the median relative infectivity per strain with 95% confidence intervals. Statistical analysis is a Welch ANOVA of direct infection with an alpha value of 0.05. *** indicates a *p*-value < 0.001. **** indicates a *p*-value < 0.0001.

To investigate the role of N-linked glycans in mediating infection by SARS-CoV-2, the seven N-linked glycans proximal to the RBD were investigated: N17, N61, N74, N122, N149, N165 and N234 (Figure 2A,B). Most of these sites are predicted to be fairly well-occupied by high-mannose or hybrid glycans, thereby increasing the likelihood that C-type lectin receptors like DC-SIGN will bind and facilitate trans-infection (Figure 2, Figures S3 and S4). Based on an analysis of over half of all PDB SARS-CoV-2 Cryo-EM structures as of 30 June 2023, the N17, N74, N149 and N165 glycans are present on highly flexible domains of the SARS-CoV-2 spike, which suggests that these glycans are on regions of the spike that are solvent-exposed or are more likely to undergo allosteric changes that could further modulate the ability of the SARS-CoV-2 spike to mediate infectivity (Figure S5). Previous literature has further predicted that glycans N165 and N234 assist in maintaining the SARS-CoV-2 spike in the RBD-up conformation, which is necessary for interaction with ACE-2 and infection [64]. 

To study the functional role of glycosylation proximal to the SARS-CoV-2 spike RBD, we created mutated SARS-CoV-2 spike genes where the asparagine (N) residues at the aforementioned glycan sites were changed to glutamine (Q) residues. All glycan mutants were created using a SARS-CoV-2 Wuhan 2019-nCoV spike (Genbank NC_045512) vector as the parent sequence. The resulting vectors were used to functionalize lentiviral pseudovirus, which was then tested on a luciferase-based infectivity assay (Figure 2C) [63]. 

To determine the effects of these mutations on direct infection, each pseudovirus containing a spike variant was used to infect HEK-293T cells transduced to stably express high levels of hACE2 (Figure 2C,D). The results showed that N17Q, N61Q and N74Q glycans had significantly reduced infectivity when compared with wild-type spike lentiviral pseudovirus, displaying a signal nearly an order of magnitude lower. This is largely in accordance with previous literature, where these glycan mutations tended to decrease infectivity about two to four fold when compared with wild-type SARS-CoV-2 spike pseudotyped virions [65,66]. The more pronounced decrease in infectivity that we observed for these mutant strains—particularly for the N74Q strain—could be explained by the fact that other systems utilized VSV-G pseudovirions (which have higher spike expression on their surface) or tested viral infectivity on different hACE2-expressing cell lines [65,66].

Oddly, the N234Q pseudovirus showed increased infectivity compared with wild-type spike lentivirus, which appears to contradict its hypothesized role in propping up the RBD in an open conformation to facilitate more efficient spike-hACE2 recognition [63]. Although unexpected, N234Q SARS-CoV-2 pseudotyped virus has been shown to have modestly decreased infectivity in the previous literature, indicating that its role as a glycan gate may not alter RBD-hACE2 recognition as much as previously thought [66,67]. 

In contrast, the N122Q, N149Q and N165Q mutants did not demonstrate any significant alteration in infectivity when compared with the wild-type SARS-CoV-2 spike. Granted, even though N122Q had too large a spread to definitively state that it caused lower infectivity, the median and mean infectivity signals for the N122Q strain did tend to be about four-fold lower than the wild-type signal. Still, these infectivity signals are largely in accordance with previous literature: either moderately decreasing infectivity (N122Q) or falling somewhere between slightly enhancing infectivity to moderately attenuating infectivity (N149Q, N165Q) [65,66]. 

**Figure 3:**
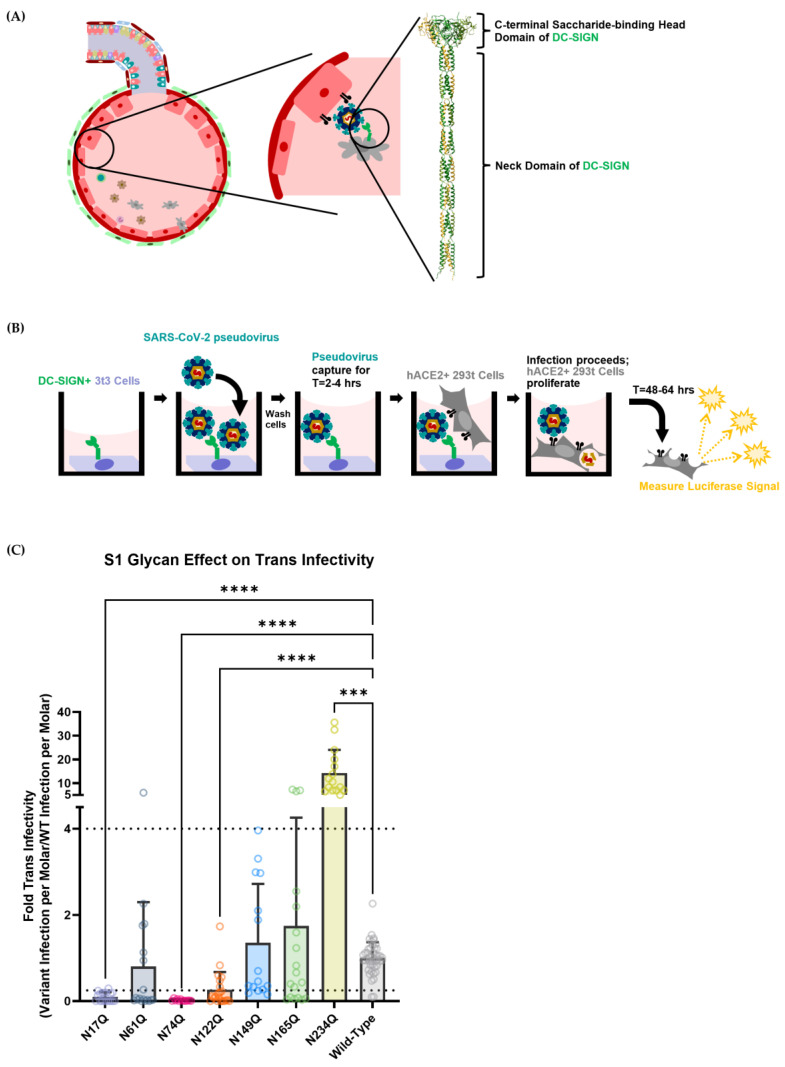
(**A**) Schematic diagram of how DC-SIGN could mediate trans-infectivity within the alveoli in vivo. On the right is an AlphaFold structure prediction of the DC-SIGN protein’s extracellular domain [68,69]. The structure was chosen by selecting the closest prediction to a previously constructed structure from Tabarani, G. et al. [70]. The DC-SIGN extracellular domain is a homotetramer that comprises a long neck domain and a C-terminal sugar-binding head domain. (**B**) Schematic diagram of the DC-SIGN-mediated trans-infection assay. In brief, 3t3 murine fibroblast cells expressing DC-SIGN were seeded into the wells of a 96-well plate. After allowing the cells to adhere, cells were incubated with SARS-CoV-2 spike pseudotyped virions for several hours before being washed. HEK-293T hACE2+ cells were then seeded over the top, allowing the captured virus to be delivered to susceptible cells, thereby modeling DC-SIGN-facilitated trans-infection. After allowing HEK-293T hACE2+ cells to undergo infection for 48–64 h, cells were lysed, and luciferase activity was measured. (**C**) Trans-infectivity of selected SARS-CoV-2 spike S1-domain N-linked glycosylation mutants. The trans-infection procedure was carried out as depicted in Figure 3B. Graphs depict median relative infectivity per strain with 95% confidence intervals. Dashed lines show a four-fold difference in infectivity, consistent with existing literature [65]. Statistical analysis was a Welch ANOVA of trans-infection with an alpha value of 0.05. *** indicates a *p*-value < 0.001. **** indicates a *p*-value < 0.0001.

### 3.2. Some SARS-CoV-2 Spike Glycans Mediate Lectin Receptor Binding and Transfer to Susceptible Cell Lines

DC-SIGN is a human lectin receptor that has already been demonstrated to bind to recombinant SARS-CoV-2 spike protein and facilitate trans-infectivity in vitro [42,43,45,67]. DC-SIGN is expressed on the surface of several immune cell subsets in the respiratory system (Figure S1). Due to the broad expression of DC-SIGN on various cell types within the respiratory system, it is accepted that DC-SIGN is a key attachment factor that allows efficient SARS-CoV-2 infection of modestly hACE2-expressing cells in the lungs (Figure 3A) [42,43,45]. 

In light of the importance of viral capture in facilitating the spread of COVID-19, the SARS-CoV-2 S1-domain glycans analyzed in Figure 2 were then assessed to measure how they contributed to DC-SIGN-mediated trans-infection. To do so, an in vitro assay was developed to isolate the process of viral capture and transfer to a permissible hACE2-expressing cell. A murine 3t3 cell fibroblast line that had been transduced with the human lectin receptor DC-SIGN (3t3 DC-SIGN+) was used to capture SARS-CoV-2 pseudotyped lentivirus and mediate trans-infection (Figure 3B). 3t3 wild-type cells and 3t3 DC-SIGN+ cells were tested to ensure they were not permissive to SARS-CoV-2 pseudovirus infection (Figure S6).

Each strain broadly followed the same trend for trans-infection signal, as for direct infection: glycans N17, N61, N74 and N122 showed decreased infectivity, while N234 showed enhanced infectivity when compared with wild-type nCoV-19 SARS-CoV-2 spike (Figure 3C). Like N122Q for direct infectivity, the N61Q strain had too large a spread to claim that it had significantly lower trans-infectivity than the wild-type virus. Although comparing the trans-infection signal of each strain to the wild-type strain gave some idea of how the glycans contribute to DC-SIGN recognition, this information does not specify whether the decrease in infectivity is due to decreased SARS-CoV-2 spike capture by DC-SIGN or whether the decrease in infectivity is due to lower hACE2-mediated direct infection. 

### 3.3. Deconvoluting Trans-Infection Propensity from Direct-Infectivity Reveals an Apparent Bias of DC-SIGN for N17 and N122

**Figure 4:**
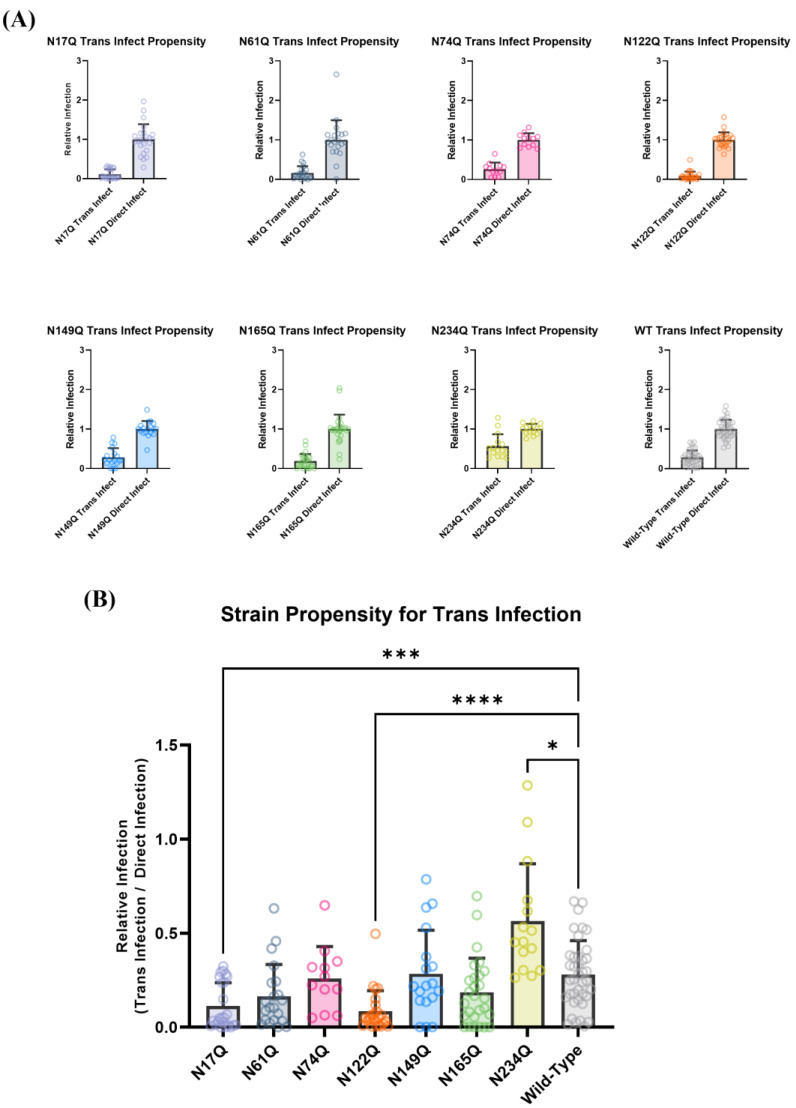
Deconvoluting the signals of SARS-CoV-2 direct and trans-infection reveals that S1-domain spike glycans are important in mediating trans-infection. (**A**) Direct- and trans- infection signals for each pseudovirus glycan variant. (**B**) Each variant’s propensity for trans-infection, where the signal of trans-infection was normalized to each variant’s hACE2-mediated direct infection. Statistical analysis was a Welch ANOVA with an alpha value of 0.05. * indicates a *p*-value < 0.1. *** indicates a *p*-value < 0.001. **** indicates a *p*-value < 0.0001.

To disentangle the effect of each glycan mutant on hACE2 entry from their effect on DC-SIGN binding, the trans-infection signal was normalized to the direct-infection signal for each SARS-CoV-2 pseudovirus strain to obtain each strain’s propensity for trans-infection (Figure 4A,B). This depicts each strain’s potential to undergo DC-SIGN-mediated trans-infection independent of the ability of each strain to bind to the hACE2 receptor. 

Wild-type SARS-CoV-2 pseudotyped virus yielded a trans-infectivity of about 28.1% of the signal of direct infection. Mutations that removed accessible glycans moderately distal to the RBD (N17Q, N122Q) significantly decreased the trans-infectivity of SARS-CoV-2 pseudotyped virions, indicating that DC-SIGN appears to primarily utilize these glycans to facilitate trans-infection. This is perhaps because these glycans are far enough away from the viral membrane to allow efficient DC-SIGN/spike protein recognition but also ensure that hACE2–RBD interactions are not sterically hindered by the carbohydrate-recognition domain of DC-SIGN itself.

Conversely, the removal of glycans that were adjacent to the RBD (N165, N61) did not appear to impede trans-infectivity. Structural analysis suggests that the DC-SIGN carbohydrate recognition domain (CRD) cannot easily access the N61 glycan because of a possible clash with the 620–641 loop region of the S2 domain of the adjacent SARS-CoV-2 spike monomer. Likewise, the DC-SIGN CRD cannot access the N165 glycan due to a possible clash with the 466–491 loop within the receptor-binding motif. Since sterically impeding the RBM region of the SARS-CoV-2 spike would drastically impair the ability of the virion to efficiently bind to hACE2, it is therefore highly unlikely that DC-SIGN is able to mediate trans-infection by binding to N165. In short, DC-SIGN has a low likelihood of binding to glycans N61 or N165, so their removal does not significantly alter these variants’ ability to undergo trans-infection. 

Interestingly, notable exceptions to this were N74 and N149: even though these glycans were distal to the SARS-CoV-2 spike RBD, the N74Q and N149Q pseudoviral mutants did not display attenuated DC-SIGN-mediated trans-infectivity, with trans-infection signals at 26.0% and 28.4% of direct-infection signal, respectively. This could be explained by the fact that DC-SIGN has a strong bias to bind to high-mannose sugars [42,43]. The consensus in the literature is that when the SARS-CoV-2 spike is expressed in mammalian cells, N74 and N149 are predominantly occupied by complex glycans, suggesting that DC-SIGN would not have high affinity for these sites and thus would not rely on these glycans to capture SARS-CoV-2 virions (Figure S3). 

Removal of N234 appeared to not only increase viral hACE2-mediated direct infectivity (Figure 2D), but also appeared to significantly increase the propensity of SARS-CoV-2 pseudotyped virus to undergo DC-SIGN-mediated trans-infection, with about two-fold higher calculated trans-infection propensity than the wild-type strain (Figure 4A,B). Even though N234 is highly occupied by mannose sugars (Figure S3)—thereby making it a favorable target for DC-SIGN binding—it is not as accessible as the other glycans, being almost completely obscured when an adjacent spike is in the down conformation. Ergo, it is probably not accessible enough to act as a reliable epitope for DC-SIGN binding. While this provides some explanation of why removal of N234 does not impair SARS-CoV-2 pseudoviral trans-infection, it does not explain the increased propensity of the SARS-CoV-2 spike for infection. 

At this point the differences in trans-infection propensity can be explained by a multitude of factors, ranging from viral instability, to virion capture by other 3t3 cell surface receptors, to DC-SIGN binding to SARS-CoV-2 spike glycans with different binding pockets [71]. Thus, it was necessary to verify that SARS-CoV-2 spike glycan recognition by DC-SIGN was the causative factor in mediating trans-infection.

### 3.4. DC-SIGN Mediated Trans-Infection Is Verified by Spike Deglycation and Mannan Inhibition

To verify that the observed differences in trans-infection were indeed due to DC-SIGN’s interaction with sugar moieties on the surface of the SARS-CoV-2 virion, we utilized a broad-acting deglycosylase called PNGase F to remove the glycans on the surface of wild-type nCoV-19-strain SARS-CoV-2 pseudotyped virions (Figure S7A). Our results appear to be in concordance with the results described in Section 3.1 and Section 3.2: the removal of glycans appeared to not only diminish trans-infection but also to diminish hACE2-mediated direct infection (Figure S7B,C). To distinguish the effect PNGase-F-catalyzed deglycosylation had on DC-SIGN-mediated trans-infection, the overall trans-infectivity signals of deglycosylated and control pseudovirus were normalized to their respective direct-infectivity signals to yield the propensity for trans-infection, analogous to what was described in Figure 4B (Figure S7D). The control wild-type pseudovirus that was not deglycosylated displayed a propensity for trans-infection of about 25–30% of the direct-infection signal, which aligned with expectations based off of the results in Figure 4B. When the virus was deglycosylated, the propensity for trans-infection was decreased to approximately 0.5–1% of the direct-infection signal. Since pseudotyped lentivirions theoretically should express only spike proteins on their surface, the results of the PNGase F test indicated that viral recognition and transfer by DC-SIGN did indeed appear to be contingent upon retaining the glycans on the SARS-CoV-2 spike protein. 

**Figure 5:**
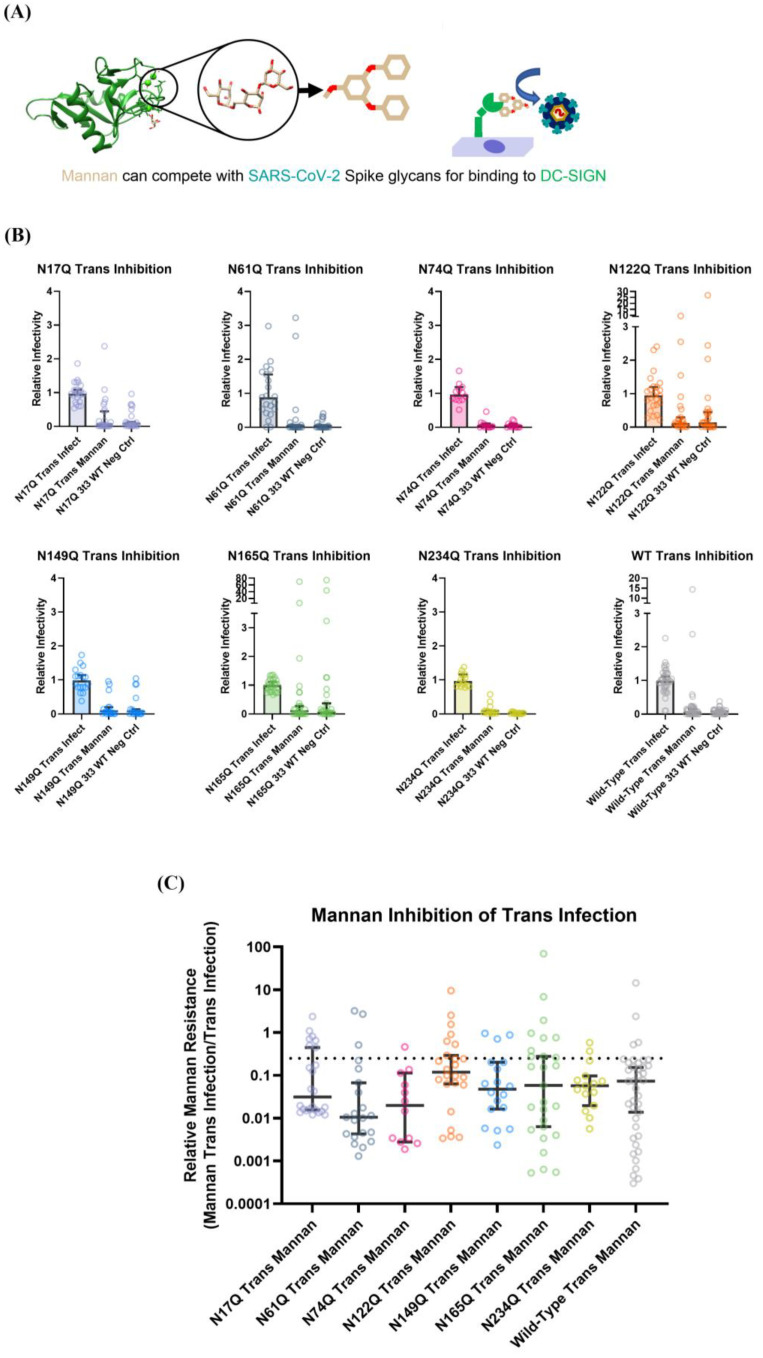
All SARS-CoV-2 pseudovirus spike glycan mutations display similar susceptibilities to mannan-based trans-inhibition. (**A**) Depiction of mannan (tan/red) acting as a competitive inhibitor of DC-SIGN (green) binding to sugars. The structure of the DC-SIGN C-terminal domain is PDB: 1SL4 according to Guo et al. (**B**) Depiction of mannan inhibition of trans-infection when compared with negative controls where 3t3 cells did not express DC-SIGN and to trans-infection assays without mannan. (**C**) Depiction of how efficiently mannan inhibited trans-infection for each glycan mutant strain. The dashed line depicts a signal four-fold decrease in infectivity, as inspired by previous literature [62]. Wild-type virus, when exposed to mannan, had its trans-infectivity reduced by 90%. A Welch and Brown–Forsythe ANOVA with an alpha value of 0.05 demonstrated that there was no significant difference in mannan inhibition between any of the strains.

Next, to assess whether specific glycans contributed more to DC-SIGN-mediated viral capture than other glycans, the trans-infection assay was performed in the presence of a competitive inhibitor of DC-SIGN binding. Mannan is a general term for a polymer of mannose sugars that can act as a competitive inhibitor of glycan–DC-SIGN binding (Figure 5A). Due to its low cost and high potency, mannan is a convenient control to validate whether the observed variation in trans-infectivity for the glycan mutant pseudoviral strains was mediated solely by DC-SIGN binding to the viral surface S1 glycans. 

First, it was verified that a concentration range of 0 μg/mL to 185 μg/mL of mannan did not significantly affect the ability of SARS-CoV-2 pseudovirions to undergo hACE2-mediated infection (Figure S8). After verifying that mannan did not appear to alter SARS-CoV-2 spike interaction with hACE2, 20 μg/mL was used to inhibit trans-infection for all SARS-CoV-2 glycan mutants. 

All SARS-CoV-2 pseudovirus spike mutant strains appeared to show identical susceptibility to mannan-based inhibition of trans-infection, with about 90–95% diminishment of signal (Figure 5B,C). This provides strong evidence that the pseudoviral strains were captured by DC-SIGN and the variations in trans-infection propensity observed in Figure 5 were indeed due to how glycan knockouts affected interactions with the DC-SIGN receptor. Ergo, our data suggest that the N-linked glycans at N17 and N122 have the highest propensity to act as binding sites for DC-SIGN to facilitate SARS-CoV-2 trans-infection. 

Given Glycan 234′s role in keeping the SARS-CoV-2 spike in the open/up conformation, it could be possible that the N234Q strain’s increased propensity for trans-infection is due to a bias for DC-SIGN to bind to the closed conformation of the SARS-CoV-2 spike [61]. This could be because of slight steric occlusion of other glycans (like N122 and N165) when the spike is in the RBD-up conformation. However, this does not explain the observed increased activity of the N234Q strain in direct-infectivity assays. Like N234, N165 is also theorized to assist in keeping the SARS-CoV-2 spike RBD in the up/open conformation [61]. While, in our work, N165Q did not make a significant change to direct infectivity, previous literature found that, in some instances, N165Q increased the hACE2-mediated-infectivity of SARS-CoV-2 pseudovirus by 20% on Calu1-hACE2+ cells [63]. Regardless of the reason, more research is needed to ascertain how the removal of these glycans is responsible for causing greater infectivity.

### 3.5. Abolishing Clusters of Glycans Severely Attenuates SARS-CoV-2 Pseudoviral Infectivity

**Figure 6:**
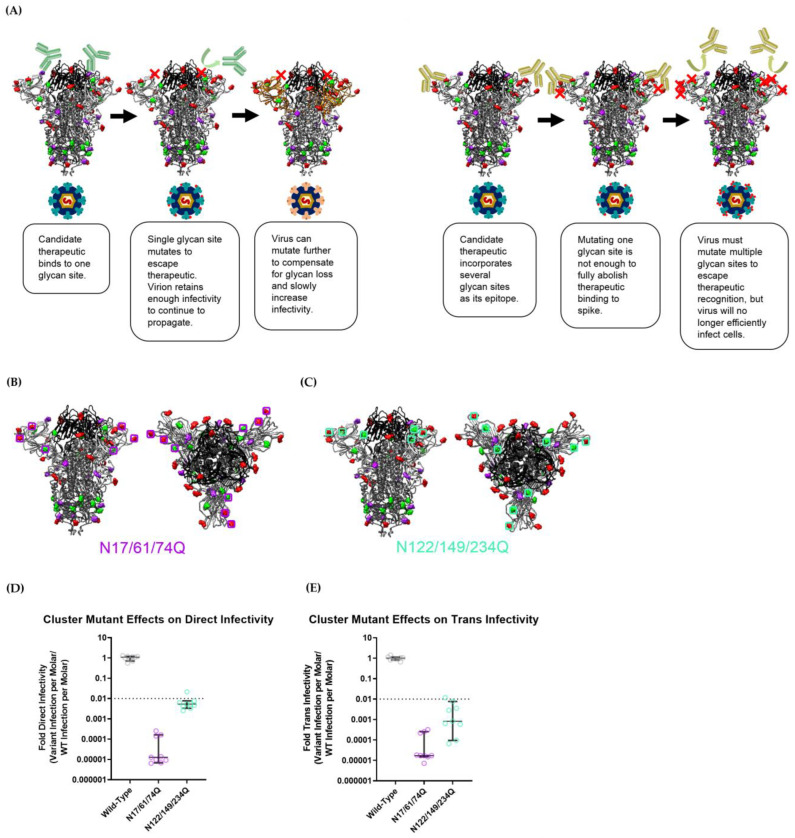
Clusters of spike glycans can act as mutation-resistant epitopes for SARS-CoV-2 inhibitor design. (**A**) Schematic display of SARS-CoV-2 mutation and immune escape. Since glycans are heavily conserved on the SARS-CoV-2 spike, it implies that they can act as robust “cold spots” for the development of therapeutics. However, SARS-CoV-2 Omicron strains have mutated significantly to escape immune detection, which implies they could further mutate to compensate for the loss of a single glycan site. Thus, it is necessary to identify clusters of glycans that will effectively eliminate viral infectivity if they are removed from the spike protein, making it far less likely for prophylactics against glycan clusters from losing efficacy against further-mutated SARS-CoV-2 strains. (**B**,**C**) Depiction of the two cluster strains that were designed: N17/61/74Q and N122/149/234Q. (**D**) Direct infectivity of cluster-N-linked glycosylation mutants normalized to wild-type/Wuhan-strain SARS-CoV-2 spike pseudovirus. (**E**) Trans-infectivity of cluster-N-linked glycosylation mutants normalized to wild-type/Wuhan-strain SARS-CoV-2 spike pseudovirus. The infectivity signal from the N17/61/74Q strain was so poor that the trans-infectivity signal was at the lower limit of detection for our plate reader/instrumentation (Figure S9A,B). (**A**–**C**) SARS-CoV-2 spike structures are adapted from PDB ID: 7NT9 by Rosa, A.

As noted, several SARS-CoV-2 spike N-terminal domain glycan mutants showed significantly attenuated pseudoviral infectivity (N17, N61 and N74 for direct infectivity and N17, N74 and N122 for trans-infectivity). These glycan sites would likely constitute “cold spots” that would be conserved due to evolutionary pressure to retain viral infectivity. Rather than relying on merely one glycan site to serve as an epitope for broad, variant-proof SARS-CoV-2 treatment, targeting S1-domain glycans as clusters could serve to provide more robust epitopes for COVID-19 vaccines and prophylactic treatments (Figure 6A). To test this strategy, two pseudoviral strains with deletions of a glycan cluster (i.e., multiple glycosylation sites in close proximity) were created: a strain with N17Q, N61Q and N74Q mutations (called N17/61/74Q) and another with N122Q, N149Q and N234Q mutations (called N122/149/234Q) (Figure 6B,C). Although Glycan N234 is not easily accessible, this particular glycan was removed to assess whether the removal of other glycans could compensate for the increased infectivity the N234Q mutant strain displayed previously. We selected N122 due to its apparent role in maintaining the strains’ trans-infectivity (Figure 4B). To complete the cluster, the glycan at N149 was selected over Glycan N165, because N149 is slightly closer to Glycan N122 than N165, and, more importantly, Glycan N149 is located on a solvent-exposed loop on the SARS-CoV-2 spike that is distal to the RBD, meaning that it could likely be leveraged as a more accessible epitope than the glycan at N165. For the purposes of our assessment, we considered a viable “cold spot” to be a glycan cluster variant that exhibits infectivity two orders of magnitude lower than the wild-type virus. This is based on existing work that shows that SARS-CoV-2 can mutate to break through prophylactics and antibodies that attenuate infectivity by 1 to 1.5 orders of magnitude [5,15,20,71,72]. Thus, we posit an epitope site that reduces viral infectivity by more than two orders of magnitude to be under sufficiently strong evolutionary pressure to not mutate. 

In a direct-infectivity assay, both cluster-mutant pseudoviral strains showed significantly reduced signals, not only compared with wild-type Wuhan nCoV-19-strain SARS-CoV-2 pseudovirus but also when compared with single-mutant strains (Figure 1 and Figure 6D). The N17Q and N74Q strains exhibited the greatest attenuation of infectivity amongst single-glycan mutants in our direct-infectivity assay, with signals of 17.9% and 3.97% of the wild-type, respectively (Figure 1). As was expected, the cluster-mutant strain N17/61/74Q—which encompassed both the N17Q and N74Q mutations—displayed a greatly diminished direct-infectivity signal, at about five orders of magnitude lower than the wild-type pseudovirus (0.00098% of wild-type) (Figure 6D and Figure S9A). For strain N122/149/234Q, the anticipated loss in infectivity from the N122Q mutation was expected to be offset to some extent by an increase in infectivity from N234Q. Ultimately, N122/149/234Q displayed more than 100-fold higher direct infectivity than N17/61/74Q, with a signal of 0.424% of the wild-type. However, this was still about an order of magnitude lower than the infectivity of the least infectious single-glycan mutant strain (N74Q) (Figure 1), and about three orders of magnitude lower than in the wild-type strain (Figure 6D), demonstrating that even though the N149Q and N234Q mutations did not impede hACE2-mediated infectivity, the removal of clusters of glycans had a combined effect attenuating SARS-CoV-2 infection. We further verified that the decrease in infectivity was not due to lower viral titer; rather, it appeared that both cluster-mutant strains had higher pseudoviral concentrations than the wild-type strain (Figure S9C). Thus, both the N17/61/74Q and N122/149/234Q glycan cluster epitopes could serve as feasible targets for anti-SARS-CoV-2 hACE2 prophylaxis. 

In terms of raw trans-infectivity, the cluster mutants N17/61/74Q and N122/149/234Q exhibited signals of 0.00152% and 0.432% of the wild-type strain, respectively (Figure 6E and Figure S9B). However, as in Figure 3, these data needed to be normalized to the direct-infectivity signal in order to ascertain the propensity for viral strains to be captured by DC-SIGN and undergo trans-infection. 

Figure S9D shows that each cluster mutant had about the same relative signal for trans-infection as direct infection. Thus, each mutant appeared to have the same propensity to undergo DC-SIGN-mediated trans-infection as the wild-type strain pseudovirus: the N17/61/74Q and N122/149/234Q strains displayed a trans-infection propensity with a median = 48.1% (avg 43.0%) and a median = 35.4% (avg 48.0%) of direct infection, respectively. For comparison, the wild-type strain exhibited a trans-infection propensity with a median = 33.8% (avg 37.0%) of direct infection.

For the N122/149/234Q strain, this behavior was expected; the increased DC-SIGN recognition observed in the N234Q single-glycan mutant strain appeared to be counteracted by the removal of some other glycan density in the N-terminal domain, perhaps bringing its trans-infection propensity down to match the wild-type strain. In contrast, the N17/61/74Q strain displayed such a diminished luciferase signal that its trans-infection luciferase reads were beneath the threshold of detection for the instrumentation (Figure S9A,B). This means that this strain could have had a significantly lower propensity for DC-SIGN-mediated trans-infection, but it simply could not be measured. At the very least, the N17/61/74Q cluster mutation did not enhance the propensity for DC-SIGN-mediated trans-infection.

Overall, these results indicate that future SARS-CoV-2 VOC strains are unlikely to mutate clusters of S1-domain glycans, since doing so would drastically diminish hACE2-mediated infectivity. Based on the aforementioned two-orders-of-magnitude threshold for infectivity, the N122/149/234 and, especially, N17/61/74 glycan clusters will likely work as robust “cold spots” for developing strain-resistant antiviral treatments for COVID-19. Specifically, we believe that a multivalent or engineered inhibitor designed to bind to N17, N61 and N74 in concert will exhibit reliable inhibitory capabilities against the numerous potential SARS-CoV-2 strains that appear in the future.

### 3.6. The Omicron BA.2 Strain May Interact with Other Cell Surface Proteins to Facilitate Trans-Infection

Over the course of the COVID-19 pandemic, the vast number of cases globally has allowed ample opportunity for mutations to arise in the SARS-CoV-2 genome, despite the relatively low mutation rate of the polymerase [10]. The GISAID database shows that every amino acid in the SARS-CoV-2 spike protein sequence has mutated at some point. While most of the mutations were not beneficial—as evidenced by their low incidence and persistence—some of these changes led to drastically increased viral fitness, typically by allowing increased infectivity or facilitating immune escape [31,46,73]. These strains were labeled “variants of interest” (VOIs) or “variants of concern” (VOCs) to highlight their clinical relevance and importance in terms of public health.

The initial Omicron SARS-CoV-2 BA.1 strain became a variant of concern primarily due to its ability to avoid detection by the host immune system [72,74,75]. It showed milder infectious characteristics in vivo when compared with other strains: Omicron BA.1 led to less damage to the alveolar spaces, spread more slowly through the lung bronchioles, and had a peak viral load in the lungs 2 days later than other strains [73]. When the Omicron BA.2 strain rapidly overtook the BA.1 strain to become the dominant VOC in mid-2022, it had mutated further to ameliorate some of the initial Omicron characteristics: Omicron BA.2 exhibited greater infectivity in the upper airways and bronchial tissues than even the wild-type or Delta-strain SARS-CoV-2 virus [76]. 

To investigate whether this drastic increase in Omicron BA.2 penetration in lung tissue could be attributed solely to greater hACE2-mediated infection, or if increased DC-SIGN binding contributed in part to the increased infectivity, Omicron BA.2 lentiviral pseudovirus was assessed in both direct- and trans-infectivity assay systems. The infectivity of Omicron-BA.2-spike pseudotyped virus was compared with several strains of COVID-19 lentiviral pseudovirus: wild-type nCoV-19 Wuhan virus, wild-type nCov-19 Wuhan virus with D614G mutation and Delta-strain pseudovirus [77]. 

**Figure 7:**
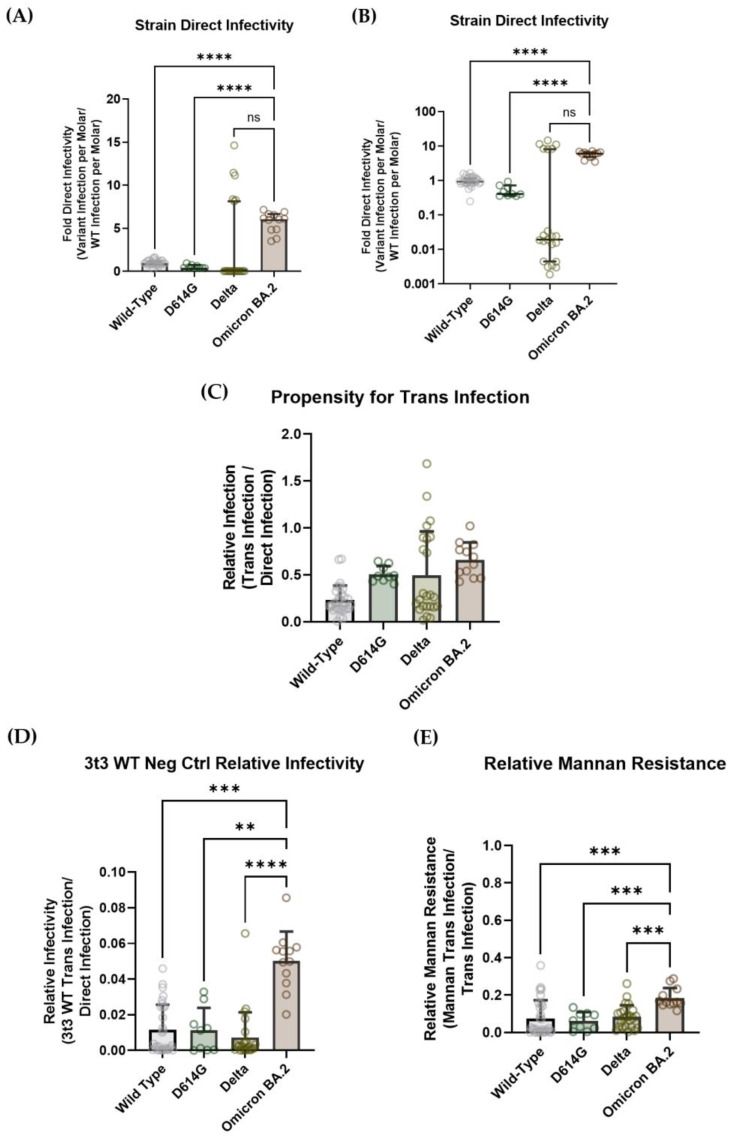
(**A**) Direct infectivity per mole for each strain of virus, normalized to wild-type/Wuhan-strain SARS-CoV-2 spike pseudovirus. (**B**) Trans-infectivity per mole for each strain of virus, normalized to wild-type/Wuhan-strain SARS-CoV-2 spike pseudovirus. (**C**) Each strain’s trans-infectivity was normalized to direct infectivity, as described in Figure 5, to obtain each strain’s propensity for trans-infection. The data show that there is no significant difference in DC-SIGN recognition between the strains. (**D**) Each strain’s trans-infection signal, when 3t3 cells that did not express DC-SIGN were used as the capture cell line. Omicron BA.2 pseudotyped virions seem to have some ability to be captured by a DC-SIGN-independent mechanism, yielding a signal that is about 0.06 (or 6%) of the direct-infectivity signal. (**E**) Each strain’s propensity to undergo mannan inhibition of trans-infection: the trans-infection signal in the presence of mannan is normalized to the trans-infection signal. The data show that Omicron BA.2 pseudovirus may have some resilience to mannan-based inhibition of trans-infection. (**A**–**E**). Statistical analysis was a Welch ANOVA with an alpha value of 0.05. ns indicates a *p*-value > 0.1, which was considered “not significant” difference between data sets. ** indicates a *p*-value < 0.01. *** indicates a *p*-value < 0.001. **** indicates a *p*-value < 0.0001.

Omicron BA.2 demonstrated significantly higher hACE2-mediated infectivity per mole compared with wild-type and D614G, with approximately six-fold more luciferase signal per mole than wild-type virus. The Delta strain displayed such a range of infectivity per mole that it was difficult to make a conclusive statistically significant statement about its propensity to undergo direct infection (Figure 7A). This is perhaps more easily depicted by comparing the raw luciferase reads for each strain to the virus titer (Figure S10). These data are largely in accordance with expectations, since, as mentioned above, Omicron BA.2 appears to infect respiratory tissue more efficiently than wild-type and Delta SARS-CoV-2 [76].

When the trans-infectivity signal was parsed from direct infectivity, the Omicron BA.2 strain did not exhibit a significant difference in trans-infection propensity when compared with the wild-type, D614G and Delta strains. As mentioned above, the Omicron BA.1 strain virus displayed altered lung infection characteristics in vivo, which led us to anticipate that Omicron strains in general might display some variation in DC-SIGN trans-infection propensity [74]. However, since Omicron BA.2 has mutated to show infection dynamics that are similar to the wild-type and Delta strains, it is plausible that Omicron BA.2 has the same propensity to undergo trans-infection as the other pseudoviral strains that were tested on our assay [76]. Furthermore, the N-linked glycosylation sites on the Omicron BA.2 spike protein are the same as in the wild-type and Delta strains, which means that all of the putative epitopes for DC-SIGN binding should be conserved (Figure 7C). 

When 20 μg/mL of mannan was added to the DC-SIGN-mediated trans-infection assay to act as a competitive inhibitor, trans-infection was decreased to about 10% for all non-Omicron strains: wild-type = 8.16%, D614G = 6.15%, Delta = 9.96%. Interestingly, Omicron BA.2 retained about twice the trans-infectivity of the other strains (21.9%) (Figure 7D). Although the Omicron BA.2 spike protein does not exhibit any mutations on its glycan sites, Omicron strains in general have about three times the number of spike mutations as other VOCs, which can cause allosteric changes that alter the conformational dynamics of the Omicron spike [7,78,79,80]. It has been shown that some Omicron-specific S2 domain mutations cause significant distortions of the post-fusion structure of the Omicron spike, such that it escapes recognition from a broadly acting peptide inhibitor [76,81]. Thus, it is conceivable that mutations in the Omicron BA.2 strain could allow conformational changes that allow the spike protein to undergo recognition by other cell surface proteins aside from DC-SIGN, thereby conferring resistance to mannan-mediated inhibition of trans-infection. Recent evidence shows that the SARS-CoV-2 spike can bind to a variety of host molecules, including neuropilin-1 (NRP1) and leucine-rich repeat-containing 15 (LRRC15) [42,82,83]. Thus, it could be possible that mutations in the Omicron BA.2 spike allow it to exhibit a higher affinity for other cell surface molecules and facilitate trans-infection mediated by other cell surface proteins. 

Overall, the observed resistance of Omicron BA.2 pseudovirus to the mannan inhibition of DC-SIGN-mediated trans-infection is likely explained by the interaction of the SARS-CoV-2 spike protein with other cell surface proteins. Indeed, performing a trans-infection assay with 3t3 cells that did not express DC-SIGN led to a similar trend as with the mannan-exposed 3t3 DC-SIGN+ cells: Omicron BA.2 can be transferred to susceptible cells slightly more than the wild-type, D614G or Delta strains (Figure 7E). Admittedly, the differences in trans-infectivity are mild, so more investigation must be done before making any claim regarding the improved ability of the Omicron BA.2 S protein to recognize and bind to alternate host factors. It is also of note that our in vitro assays (where a susceptible cell line is added over the top of a ~20% confluent cell monolayer used to capture pseudovirions) do not replicate the environment of lung tissue in the context of true COVID-19 infection. 

## 4. Discussion

Over the course of the past 3 years, the COVID-19 pandemic has shown that SARS-CoV-2 (and Beta family coronaviruses in general) is incredibly contagious, going from a localized epidemic in Wuhan to a global pandemic in the span of 4 months. The high infectivity of the SARS-CoV-2 virus allows it ample opportunity to mutate, as evidenced by the rise of several breakthrough strains that overcame existing therapeutics and vaccines [4,5,7,8,11,62]. Coupled with this, the high transmissibility and mutation rate of COVID-19 has allowed it to make multiple zoonotic jumps during its relatively young existence: in addition to its accepted origins as a pangolin coronavirus (originally derived from RatG13, a horseshoe bat coronavirus), SARS-CoV-2 has already jumped from humans to other mammal species like mink and deer, causing concern that the virus could undergo unforeseen mutations in another animal population before jumping back to humans [10,32,84]. Hence, there is still a pressing need to identify immutable epitopes on the SARS-CoV-2 virion—also known as “cold spots”—to act as targets for the creation of vaccines and prophylactics that can continue to efficiently inhibit future variants of SARS-CoV-2. Since SARS-CoV-2 establishes an initial infection in the host’s respiratory system, this work has a particular focus on identifying epitopes that are involved in the unique process of infecting lung tissue, as opposed to solely focusing on areas of virus that contribute to canonical hACE2-mediated infection (also called “direct infection” in this paper’s parlance). 

In humans, penetration into the proximal airways of the nasal cavity is the critical step of establishing replicative SARS-CoV-2 infection [85]. Particularly, the ciliated epithelial cells of the nasal and proximal airways express sufficiently high levels of hACE2 to allow efficient viral entry and SARS-CoV-2 infection [86,87]. Research has shown that the respiratory airways exhibit a gradient in hACE2 expression, whereby the distal airways deeper in the lungs have much lower hACE2 expression than the proximal and nasal airways [62,87,88]. Despite the low hACE2 expression of the distal airways, the clinical outcomes of COVID-19 disease have consistently shown that SARS-CoV-2 infection damages the lung tissue, sometimes even in lieu of systemic infection with SARS-CoV-2 [49,85,88,89,90,91,92,93]. Specifically, COVID-19 presents with dysregulation of surfactant-producing Type-2 alveolar pneumocyte cells; this results in deterioration of the function of the respiratory system as the alveoli collapse and no longer allow gas exchange [89,90,91,94,95]. Autopsy studies clearly show cytopathic changes in Type-2 alveolar cells post-mortem, in spite of the fact that no subset of distal lung cells show over ~6% hACE2 expression within the population [88,89,91,94,96,97,98]. 

At the start of 2021, it was still unknown exactly how SARS-CoV-2 was able to reliably infect the cells of the respiratory tissue [50]. Soon thereafter, researchers analyzed cell expression databases and found that C-type lectin transmembrane proteins are expressed on the surface of a variety of epithelial, adventitial and resident immune cells of the lungs [42]. Cell surface lectin receptors have been demonstrated to bind to glycans on the surface of SARS-CoV-2 glycoproteins and facilitate efficient trans-infection to distal lung airway cells and cells within the alveoli, effectively enhancing the infectivity of SARS-CoV-2 in the lung airways [39,40,42,44]. This process of viral protein glycosaminoglycan-mediated trans-infection has been well-characterized for other enveloped viruses, such as HIV, dengue virus, West Nile virus, Ebola virus and more [38,39,40]. Ergo, it is accepted that lectin receptors are responsible for facilitating efficient infection of cells in the respiratory system [42,43,44,49].

In this study, we assessed the effects of select spike S1-domain glycans and SARS-CoV-2 spike proteins from different strains in mediating viral infectivity on in vitro assays that modeled both hACE2-mediated direct infection and C-type lectin-receptor-mediated trans-infection [10]. Specifically, the C-type lectin DC-SIGN was used as the viral capture receptor, since it has been shown to be capable of binding SARS-CoV-2 spike proteins in microscopy assays and facilitating virion transfer to cells that modestly express hACE2 [42,43].

Unlike other viruses that readily shift glycan sites on their spike glycoproteins to assist in evading immune detection, SARS-CoV-2 displays a remarkable degree of immutability regarding its glycan pattern [38,39,40]. Out of the 101,461 SARS-CoV-2 spike glycoprotein sequences that were deposited in the NCBI Virus Database in the past six months, less than 0.3% show any mutations at any of the 22 asparagine residues that are consistently shown to be glycosylated [34,35,36,37,99]. Furthermore, even if a strain does exhibit a mutation at a glycan site, evidence suggests that the strain will quickly be outcompeted: in early 2022, some samples of Omicron BA.2 from patients in the UK exhibited an N74K mutation, but a mere month later this mutation was no longer found in the population [19]. In another case, Omicron CM.1 is currently the only VOC strain on the NCBI Virus Database that shows a mutation at any spike protein glycan site (N17S) [99]. While multiple sequences of Omicron CM.1 were detected in Ireland, Switzerland, California and Texas, there has only been one recorded case of Omicron CM.1 reported globally in the past 6 months [99]. Although Delta B.1.617.2 and several emerging Omicron XBB strains of SARS-CoV-2 do have mutations at T19 that impede glycosylation at the N17 sequon, it is known that oligosaccharyltransferases can tolerate some degree of mutation and still attach sugars to asparagines, suggesting that N17 could still be glycosylated in some strains despite the mutation [100,101]. Regardless, the maintenance of glycan sites in the vast majority of VOC and VOI strains implies that SARS-CoV-2 virions require their N-linked glycan sites in order to retain infectivity and are thus viable targets for COVID-19 prophylactic development. 

Since the majority of prophylactic antibodies and vaccines recognize the S1 domain of the spike protein, we focused our analysis on uncovering the importance of the glycans proximal to these antibody epitopes [22,75,102]. We avoided assessing glycans on the spike receptor-binding domain (such as N331 and N343) because many existing prophylactics already recognize the RBD and we wished to identify novel epitopes that could act in combination with existing therapeutics. To that end, glycans N17, N61, N74, N122, N149, N165 and N234 were selected for analysis.

Our analysis showed that the removal of N17, N61 and N74 significantly impaired SARS-CoV-2 pseudovirions in terms of hACE2-mediated infection, while the removal of the glycans N149 and N165 had no effect (Figure 2D). This is largely in accordance with existing literature, as N17Q, N61Q, N74Q and N122Q have all been reported to either significantly attenuate infectivity or have no effect [66,85]. On the other hand, glycans N149 and N165 have been shown to have unpredictable effects on hACE2-mediated infection of SARS-CoV-2 pseudotyped viruses, ranging from modestly increasing infectivity to significantly decreasing infectivity [66,85]. Ergo, it is likely that the different effects of N149Q and N165Q are more likely due to idiosyncrasies of the infectivity assays (e.g., using lentiviral pseudovirions versus VSV pseudovirions, or using different susceptible cell lines) rather than a consistent effect of glycan mutations in altering SARS-CoV-2 spike dynamics or fusogenic activity. 

In our work, the removal of N234 appeared to increase the ability of pseudovirions to display hACE2-mediated direct infectivity. While this is unexpected, given the predicted role of N234 in propping up the spike RBD in the infectious “up” conformation, previous studies have shown that N234 knockout mutations do not affect virion infectivity as much as the purported role of N234 would imply [65,66]. Indeed, mutating N165—which was hypothesized to perform a similar role to N234—also appeared to boost the infectivity of pseudovirions in previously published literature [64,66]. 

Lectin-receptor-mediated trans-infectivity is arguably more important in dictating the initial stages of COVID-19 infection in patients due to the modest hACE2 expression of resident lung cells [42,50,51,52]. Our hope was that the selected mutated glycan epitopes could provide the basis for a new class of COVID-19 inhibitors that could work in combinatorial therapies without impeding anti-hACE2 prophylactics. To test this, we developed an in vitro assay that could mimic the process of DC-SIGN-mediated SARS-CoV-2 pseudoviral capture and presentation to a hACE2+ cell line (Figure 3A,B). After running these assays, each strain’s trans-infectivity signal was normalized to its direct infectivity to obtain the propensity of each glycan mutation for mediating DC-SIGN attachment and transmission to permissive hACE2+ cells (Figure 4B).

After comparing the propensities of glycan mutant pseudovirus strains, glycans N17 and N122 both appeared to be the most integral in enabling DC-SIGN-mediated trans-infection. The locations of N17 and N122 on the spike protein seem to indicate that DC-SIGN has a preference for binding glycans that are not too close to the RBD, but are also not completely on the periphery of the S1 domain of the spike (Figure 2A,B). The importance of N17Q in mediating both direct and trans-infection may have been implied by the course of the COVID-19 pandemic: several strains of SARS-CoV-2 that showed mutations at the N17 sequon have been quickly outcompeted by other strains [103]. However, this argument is somewhat invalidated by the fact that N17 is also predicted to be predominantly occupied by complex glycans, which should mean that DC-SIGN would not have high binding affinity for the N17 glycans. This may provide some credence to the idea that allosteric and conformational variations make certain spike glycans particularly vulnerable to DC-SIGN recognition and that S1-domain glycans that are neither proximal to the RBD nor present on the periphery of the SARS-CoV-2 spike are uniquely suited to facilitate DC-SIGN-mediated trans-infection (Figure 2A,B and Figure 3C). Alternatively, virus-expressed spike proteins tend to have less complex glycosylation at each SARS-CoV-2 spike N-linked glycan site than mammalian-expressed soluble spike [104]. This may mean that the N17 glycans on virions are high-mannose and thus truly do serve as viable epitopes for DC-SIGN-mediated trans-infection. Finally, another possible factor to consider is DC-SIGN’s high affinity for fucosylated glycans [105]. Fucose is a 6-deoxy hexose that is occasionally added onto branched N-linked glycans (both complex and hybrid glycans) and also onto O-linked glycans [105,106,107]. Existing literature has shown that the SARS-CoV-2 spike protein glycans N17, N74, N149 and N165 can have some degree of fucosylation, meaning that the glycans we identified to be integral in facilitating DC-SIGN-mediated trans-infection could have simply been sites that have a high propensity to undergo fucosylation [108]. For future experiments, it would be prudent to perform a complete assessment of glycosylation patterns on the SARS-CoV-2 virus, including assessing how fucosylation patterns differ between spike proteins that are made in workhorse immortalized lab cell strains versus spike proteins that are made in lung tissue cell lines.

Unlike the other glycan mutations, N234Q pseudovirions displayed an increased propensity to be captured by DC-SIGN, as evidenced by the N234Q strain’s increased propensity for trans-infection while still having the same susceptibility to mannan inhibition of DC-SIGN-mediated trans-infection as other strains (Figure 4B and Figure 5C). Given that N234 is predicted to aid in keeping the SARS-CoV-2 spike in the RBD up/open conformation, this leads us to wonder whether the closed conformation of the SARS-CoV-2 spike has a greater affinity for DC-SIGN than the open conformation [64]. Like N234, N165 is also predicted to assist in propping the RBD of the SARS-CoV-2 spike in the up position. While we did not observe a similar increase in trans-infectivity for the N165Q strain, it did not display significantly attenuated trans-infectivity either. This could be explained by the fact that the N165 glycan is predicted to have less hydrogen bonding to prop the RBD in the up position, which suggests that removal of N165 may not have altered the conformational dynamics of the SARS-CoV-2 spike enough to alter the ability of DC-SIGN to bind [66]. More work is needed to verify the effects of N234 and N165 on the dynamics of the SARS-CoV-2 spike, with a possible need to ascertain whether N234Q/N165Q double mutants would no longer have the ability to retain the up/open conformation of the spike protein. Alternatively, it could be possible that the RBD-down/closed conformation of the SARS-CoV-2 spike exposes high-mannose glycans to facilitate easier trans-infection. Thus, another potentially important path of scientific inquiry is to test whether DC-SIGN and other attachment receptors have higher affinity for the RBD-down/closed conformation of the SARS-CoV-2 spike than the RBD-up/open conformation.

Interestingly, our results do not seem to agree with FACS-mediated protein–protein binding data from a preprinted article: the authors reported that, out of all of the glycans, only the removal of glycosylation at N149 was observed to alter DC-SIGN binding to the SARS-CoV-2 spike protein, with N149Q decreasing binding by about two orders of magnitude [109]. Overall, the lack of consensus in the literature suggests that more work is needed to fully grasp what roles of individual S1-domain glycans are most important in mediating infection. 

Omicron-family virus variants have become the dominant circulating strains, exhibiting effective breakthrough infections despite displaying lower fusogenic activity than not only the Beta and Delta variants of SARS-CoV-2 but also the wild-type nCov-19 Wuhan virus [6,74,110]. What is most concerning regarding the rise of Omicron variants is their immune evasion characteristics, demonstrating that SARS-CoV-2 can mutate relatively quickly and tolerate significant alterations on its spike protein to regain the ability to efficiently infect humans. The current literature shows that Omicron SARS-CoV-2 was able to establish breakthrough infections when convalescent sera or antibody treatments were diluted by 1 to 1.5 orders of magnitude in in vitro assays [4,5,15,20,72,74,75,102]. Therefore, we considered that the threshold for identifying a viable SARS-CoV-2 spike “cold spot” was an epitope that would lower pseudoviral infectivity by at least two orders of magnitude when compared with wild-type nCov-19 Wuhan pseudovirus. This was done so that if escape mutants started to develop to remove the “cold spot” epitope, the resultant strains would lose so much infectious activity that they would be unable to establish the persistent infection of a host. 

With the advent of combinatorial prophylactic treatments like bispecific antibodies, chimeric viral entry inhibitors and de novo inhibitors designed in silico, it is relatively easy to develop a prophylactic therapy that targets discrete sites on a target virus. Given the inherent evolutionary pressure for SARS-CoV-2 to avoid mutating its N-linked glycosylation sites, we hoped to identify viable glycan clusters that could serve as highly conserved epitopes that could act as “cold spots” to ensure that SARS-CoV-2 infection did not mutate for further mutant strains. The removal of three glycans in tandem drastically decreased the infectivity of our pseudovirions, with even the strain including N234Q (which consistently appeared to increase infectivity) demonstrating infectivity three orders of magnitude lower than unmodified spike virions. There are already prophylactic strategies against other viruses that leverage binding to glycans on the viral spike protein: several anti-HIV antibodies bind to the glycan shield rather than amino acid epitopes, and other globular-lectin-protein-based inhibitors such as griffithsin exhibit broad inhibitory activity against a variety of viruses [54,111,112,113]. Recent literature has shown considerable success when griffithsin treatment was combined with a therapeutic that targeted a different epitope on the SARS-CoV-2 spike—a designed viral fusion inhibitor called EK1, sulfated polysaccharides called carrageenan, or another glycan-binding inhibitor called cyanovirin [114,115,116,117]. These results hint that if new inhibitors could be designed against unique SARS-CoV-2 epitopes (such as the N17/61/74 or N122/145/234 glycan clusters), they could be combined with other existing COVID-19 therapeutics to create a more potent prophylactic treatment. Furthermore, SARS-CoV-2 virions also contain the M, N and E structural proteins. It has already been established that addition of the N protein can significantly increase the hACE2-mediated infectivity of SARS-CoV-2 pseudovirions [60]. Since M and E proteins are glycosylated, membrane-embedded proteins, and tend to not mutate as frequently as spike protein, there is a distinct possibility that these proteins could also act as target “cold-spot” epitopes for combinatorial anti-transinfection therapy [118]. Current work from our group is ongoing to ascertain whether these structural proteins can potentiate trans-infectivity and therefore be included [article in preparation].

At this point, it has been well demonstrated that Omicron strains of COVID-19 lead to significantly different infection dynamics in the lungs than previous strains: the Omicron strain tends to reach peak lung tissue infectivity several days post-infection, possibly indicating that the Omicron virus has a longer dwell time in the lung airways, mediated by virion capture by attachment receptors, before undergoing hACE2-mediated infection [74,110]. Given that trans-infection is theorized to be a significant contributor to how SARS-CoV-2 establishes infection in the host respiratory system, it was prudent to assess how spike proteins from several variants of concern could alter the ability of pseudovirions to undergo DC-SIGN-mediated infection. D614G, Delta and Omicron BA.2 spike proteins were all tested on both direct and trans-infection assays. In our work, D614G and Delta did not show significantly increased propensities for undergoing DC-SIGN-mediated trans-infection, which was not unexpected considering the relatively few mutations on the spike protein for these strains. It was surprising that D614G and Delta pseudovirus strains exhibited only slightly higher hACE2-mediated infection compared with wild-type spike pseudotyped lentivirus, considering that existing literature shows Delta-strain SARS-CoV-2 causes more severe infection [12]. 

In our work, the Omicron BA.2 pseudovirus strain showed significantly higher hACE2-mediated infectivity than the other tested strains. Recent literature has shown that Omicron strains range from having comparable infectivity to wild-type SARS-CoV-2 to having higher infectivity than the D614G and Delta strains [12,15,76]. This variation is likely due to the intricacies of the viral systems and susceptible cell lines that are used for testing the SARS-CoV-2 spike strains; even different airways in the lungs (i.e., nasal, proximal, distal) have varying susceptibilities to different variants of SARS-CoV-2 [12,76]. Thus, while we observed greater direct infectivity for Omicron BA.2 in hACE2-expressing HEK-293T cells, we are cautious in making any claims regarding Omicron-BA.2-strain SARS-CoV-2 having higher infectivity in the context of infecting humans, especially considering the complexity of in vivo tissues.

There is mounting evidence that numerous mutations of the Omicron strain cause allosteric changes in the spike protein [76,78,79,81]. Because the SARS-CoV-2 spike has been shown to bind to a multitude of cell surface molecules—including sialic-acid-binding surface receptors, heparan sulfate, glycosphingolipids and more—it is plausible that the predicted unique structural dynamics of the Omicron BA.2 strain spike could confer resistance to mannan-mediated inhibition of trans-infection, as well as some ability to be captured by a fibroblast cell line that did not express DC-SIGN [42,82,83]. Most notably, Omicron mutations lead to greater spike processing by furin, which is known to reveal an epitope that is capable of being bound by the cell surface receptors neuropilin-1 (NRP1) and neuropilin-2 (NRP2) [119,120]. 

Several SARS-CoV-2 VOCs have shown mutations close to the furin cleavage site, with H655Y and P681H each individually increasing the propensity of the spike protein to undergo furin processing [121,122]. Omicron strains all display both mutations, and they appear to undergo slightly increased spike processing in vitro [121]. The 3t3 DC-SIGN cells that acted as the capture cell line in our trans-infection assay were murine fibroblasts, which are known to express high levels of the NRP1 homolog NRP2 [120,123]. Ergo, it is possible that the Omicron BA.2 pseudovirus’s resistance to mannan inhibition of DC-SIGN-mediated SARS-CoV-2 trans-infectivity is due to enhanced furin cleavage, and thus increased attachment to neuropilin-2.

## 5. Conclusions

Overall, this work focused on assessing several S1-domain spike glycans, and three VOC strain spike proteins could affect the ability of SARS-CoV-2 to establish initial infection in the host lung tissue. Since respiratory tissue only modestly expresses hACE2, it is widely accepted that virion capture and trans-infection is the chief process in facilitating infection of the distal airways. Ergo, particular interest was given to testing how the aforementioned characteristics of the SARS-CoV-2 virion can affect DC-SIGN-mediated trans-infection. 

As would be expected from previous literature, the removal of most of the S1-domain glycans (specifically, N17, N61 and N74) decreased the hACE2-mediated direct infection of the SARS-CoV-2 pseudovirions. The effect of glycan removal on trans-infectivity was more nuanced, with only the removal of the N-linked glycans at N17 and N122 significantly impairing the trans-infectivity of SARS-CoV-2. Interestingly, the removal of the glycan at N234 increased both the direct infectivity and trans-infectivity of the pseudotyped virions, calling into question the hypothesized role of the N234 glycan in ensuring the SARS-CoV-2 spike stays in the RBD-up infectious conformation. 

Given that SARS-CoV-2 Omicron strains have mutated significantly to evade immune detection, eventually leading to strains that are capable of establishing breakthrough infections in patients who have received multiple doses of COVID-19 vaccines, we considered whether glycan cluster sites could act as epitopes for the development of prophylactic treatments. The priority was to identify sites that were highly unlikely to mutate due to the evolutionary pressure to maintain SARS-CoV-2 virus infectivity. Our work found that the glycan cluster mutations N122/149/234Q and N17/61/74Q both significantly impaired SARS-CoV-2 pseudotyped virus, with infectivity about two orders of magnitude and five orders of magnitude lower, respectively. 

Finally, we assessed whether different strains of SARS-CoV-2 could affect how pseudotyped virions undergo trans-infection. As expected based on existing literature, in our work the Omicron BA.2 strain exhibited increased hACE2 direct infectivity when compared with the wild-type strain [124]. While there was no difference in the strains’ ability to undergo DC-SIGN-mediated trans-infection, our results hint that Omicron BA.2 may have a slightly increased propensity to bind to alternative attachment receptors, like neuropilin-2.

Although we found some key facets of the SARS-CoV-2 spike protein that appeared to affect both hACE2-mediated direct infectivity and DC-SIGN-mediated trans-infectivity, we finish by emphasizing that our experiments were performed in immortalized lab-adapted cell lines. Optimally, we believe it is important to verify the effects of spike protein glycan mutations and the Omicron BA.2 virus strain on an ex vivo model of human respiratory tissue.

## Figures and Tables

**Table 1:** Mutation Rates at N-linked Glycan Asparagine Residues on the SARS-CoV-2 Spike Protein as of August 2023 [57].

N-Linked Glycan Location	Proportion of CoV-GLUE-Parsed GISAID Sequences with Mutations at Site (Substitution, Insertion and Deletion Mutations)
N17	0.00057
N61	0.000251
N74	0.000284
N122	0.000151
N149	0.000136
N165	0.000067
N234	0.000135
N282	0.00013
N331	0.000055
N343	0.000012
N603	0.000095
N616	0.000146
N657	0.000094
N709	0.000062
N717	0.000085
N801	0.00005
N1074	0.00292
N1098	0.000022
N1134	0.000051
N1158	0.000048
N1173	0.00008
N1194	0.000046

## Data Availability

Data available on request.

## Supplementary Materials

The following supporting information can be downloaded at https://www.mdpi.com/article/10.3390/v15091901/s1. Figure S1: Simplified depiction of hACE2- and DC-SIGN-expressing cell types in the distal airway and alveolus of the lung, with a particular focus on immune cells; Figure S2: SARS-CoV-2 spike gene mutations for key VOC and VOI strains, as specified by the European Centre for Disease Prevention and Control, the World Health Organization and the SIB Swiss Institute of Bioinformatics’ ViralZone Website, and an analysis of residue mutation rates based off of a database of SARS-CoV-2 sequences [57]; Figure S3: Glycan occupancy of the SARS-CoV-2 spike, based on an average of existing literature [34,35,36,37,66,103,104,108,125,126,127,128,129,130,131]; Figure S4: Estimated glycan occupancy of the SARS-CoV-2 spike in existing literature, displayed separately [34,35,36,37,66,103,104,108,125,126,127,128,129,130,131]; Figure S5: Estimation of spike flexibility at N-linked glycan sites based on an analysis of PDB cryo-EM structures of the SARS-CoV-2 spike; Figure S6: Verification that murine fibroblast 3t3 wild-type and 3t3 DC-SIGN+ cell lines are not susceptible to SARS-CoV-2 infection.; Figure S7: Depiction of the deglycosylation of pseudoviral samples and data showing that deglycosylation decreases both direct and trans-infectivity; Figure S8: Assessment of whether mannan affects the direct infectivity of the SARS-CoV-2 pseudovirus; Figure S9: Raw infectivity data for the SARS-CoV-2 pseudotyped lentivirus cluster-mutant strains N17/61/74Q and N122/149/234Q; Figure S10: Raw infectivity data and virus titer for the SARS-CoV-2 wild-type Wuhan-nCov, D614G, Delta and Omicron BA.2 strains of pseudotyped lentivirus.
