# Supplementary material for: The Effect of Select SARS-CoV-2 N-Linked Glycan and Variant of Concern Spike Protein Mutations on C-Type Lectin-Receptor-Mediated Infection
Source: Viruses. 2023 Sep 9;15(9):1901. doi: 10.3390/v15091901 (PMC10535197; doi:10.3390/v15091901)
Supplement: Supplementary file 1 [file viruses-15-01901-s001.zip › viruses-2557296-Supplemental.pdf]

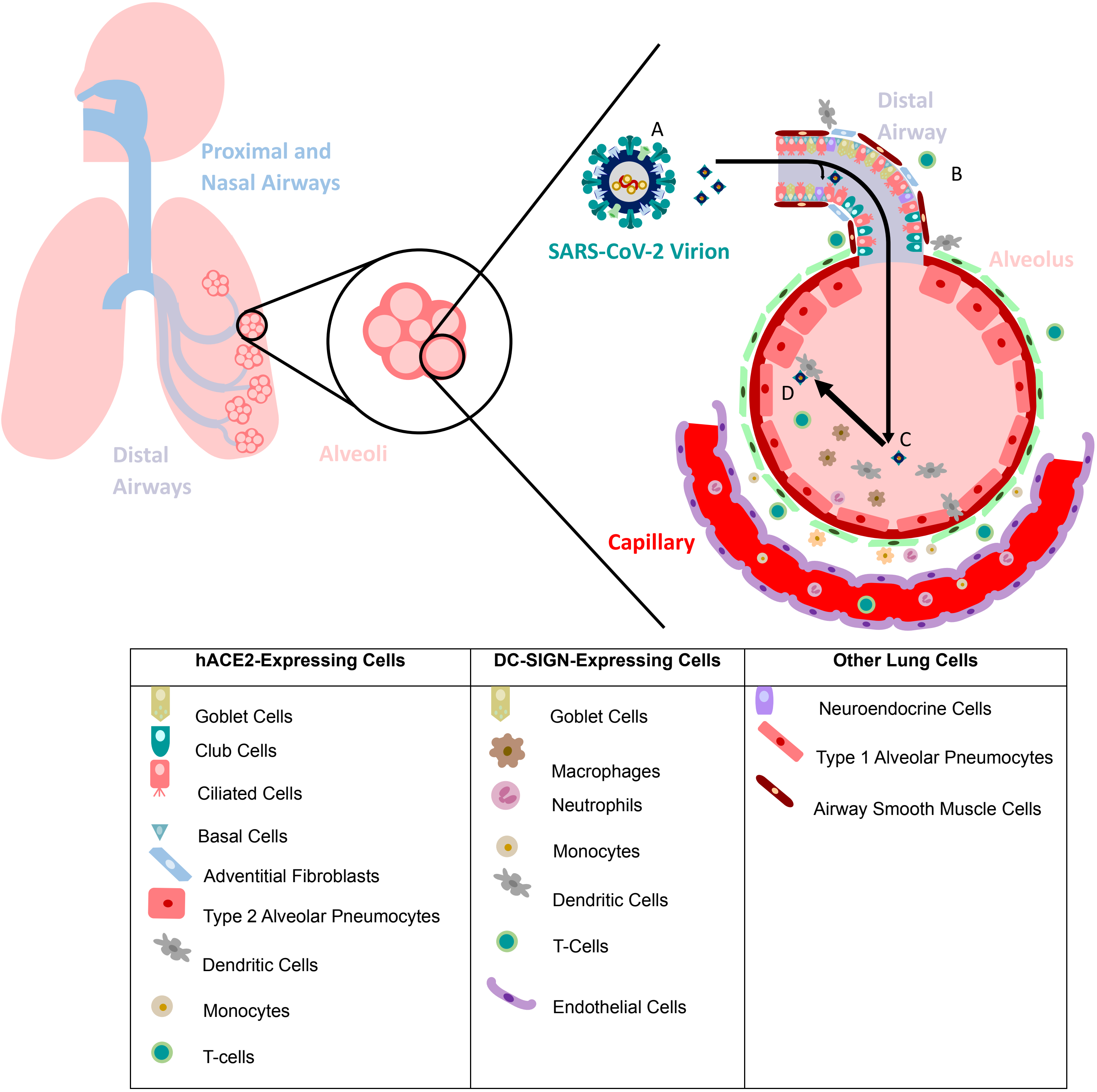

Figure S1. Simplified depiction of hACE2 and DC-SIGN expressing cell types in the distal airway and alveolus of the lung, with a particular focus on immune cells. SARS-CoV-2 virions (A) can infect the squamous epithelial cells of the nasal and upper respiratory airways (not shown), or be conducted to the distal airways, which are comprised of specialized columnar epithelial cells. Some of these cells express enough levels of hACE2 to allow for virion entry (B). But only a small subset of cells in the distal airways express DC-

SIGN, meaning that infection of the distal airways isn't very efficient. Most SARS-CoV-2 virions enter into the alveolus and encounter the resident DC-SIGN expressing immune cells (C). These DC-SIGN expressing cells facilitate viral infection-in-trans to hACE2-expressing cells, such as Type 2 Alveolar Pneumocytes (D).

(A)

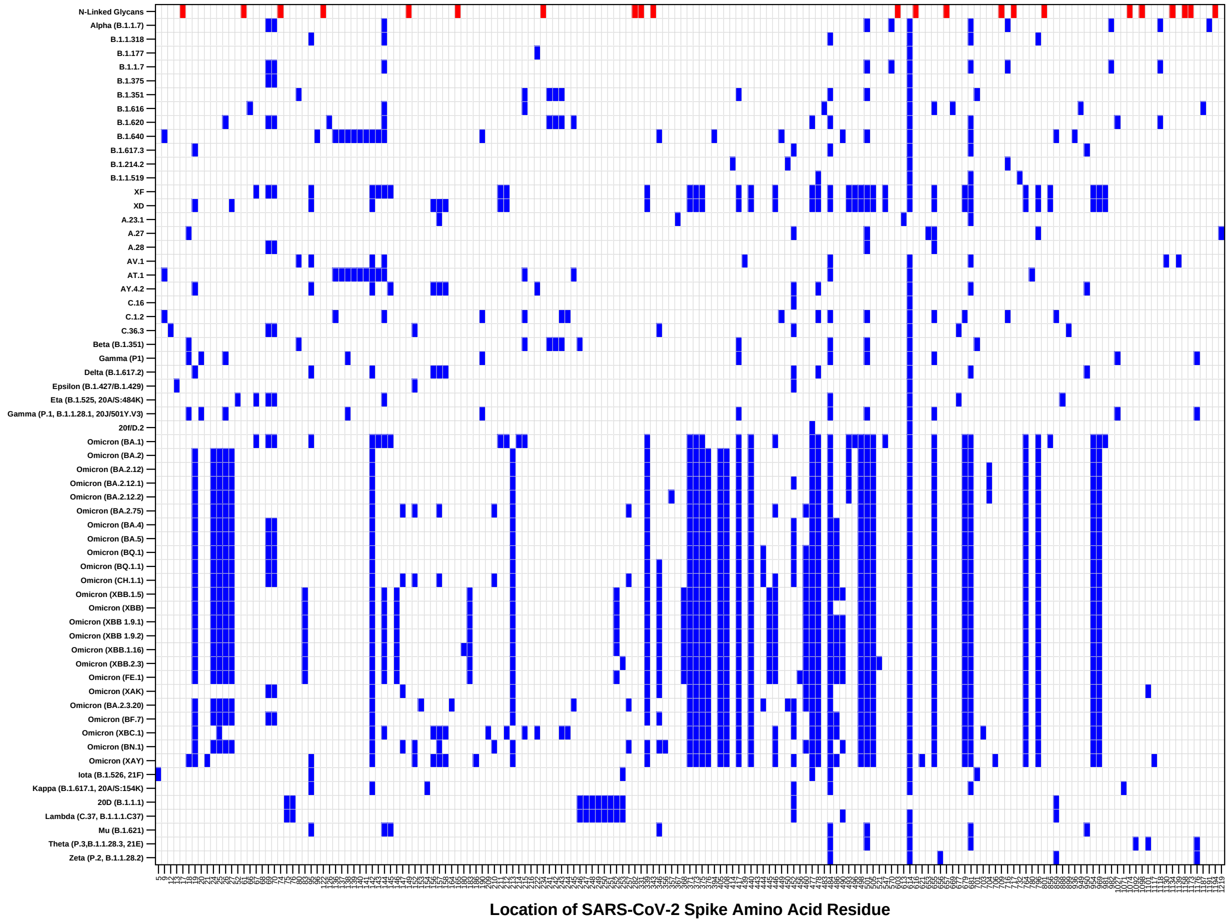

(B)

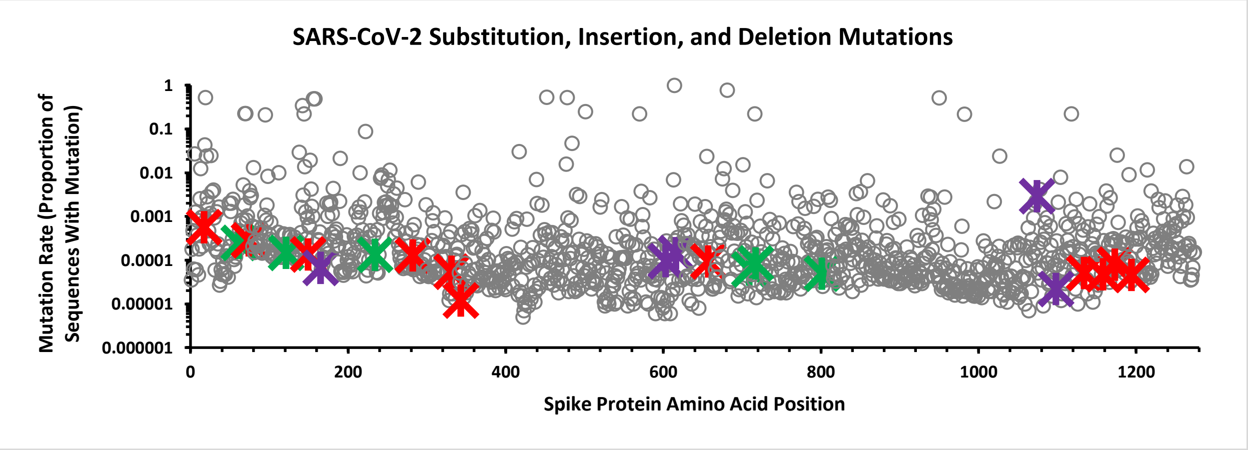

Figure S2. (A) Depiction of SARS-CoV-2 Spike gene mutations for key VOC strains, as specified by the European Centre for Disease Prevention and Control (https://www.ecdc.europa.eu/en/covid-19/variants-concern), the World Health Organization (https://www.who.int/activities/tracking-SARS-CoV-2-variants), and the SIB Swiss Institute of Bioinformatics' ViralZone Website (https://viralzone.expasy.org/9556). Glycan sites are

depicted at the top row, in red. Location of mutations found in specific Covid-19 viral strains are depicted in blue. **(B)** Depiction of the mutation rate (substitution, insertion, and deletion mutation) at each residue of the SARS-CoV-2 Spike protein as based on an analysis of a curated database of SARS-CoV-2 gene sequences [57]. Red, Green, and Purple Ж crosses depict locations of N-linked glycans, as specified in Figure 1. We want to stress that these data report on whether there has been a verified sequence that has a mutation at a particular residue site; this includes sequences that had mutations that were rapidly outcompeted by non-mutated strains. In other words, this data does not take into account the loss of fitness that mutations could cause.

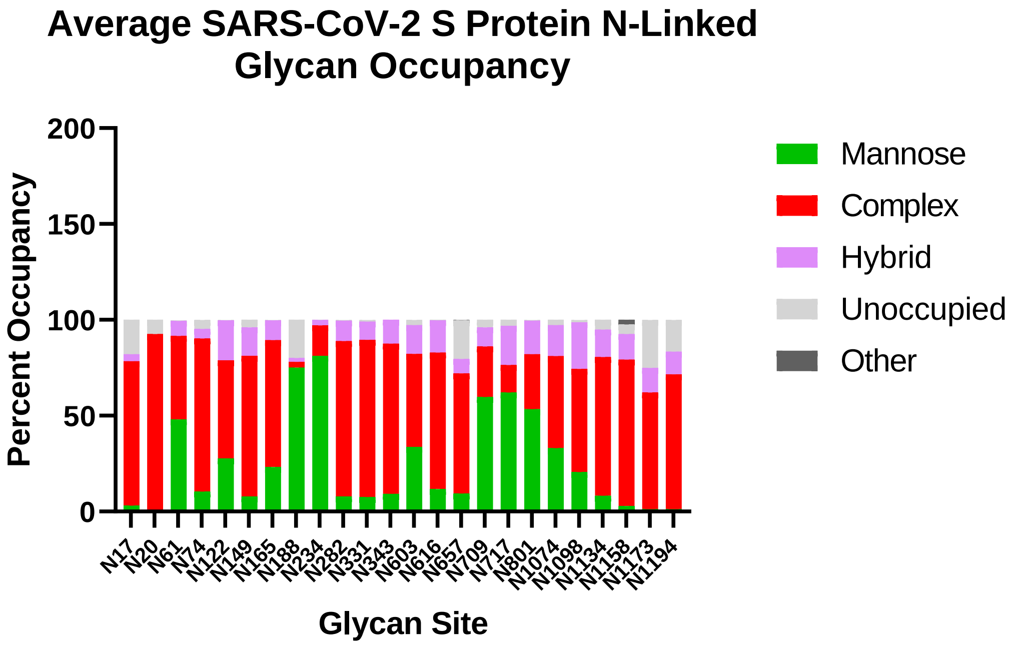

Figure S3. The glycan occupancy of the SARS-CoV-2 Spike based on an average of existing literature. For those papers that did not report the exact values, the glycan occupancy was estimated based off of an analysis of the .png figures using the Plotdigitizer application (https://plotdigitizer.com/). Note the presence of glycans N20 and N188, both of which are the result of mutations in the Spike protein sequence of the Gamma P.1 strain [128].

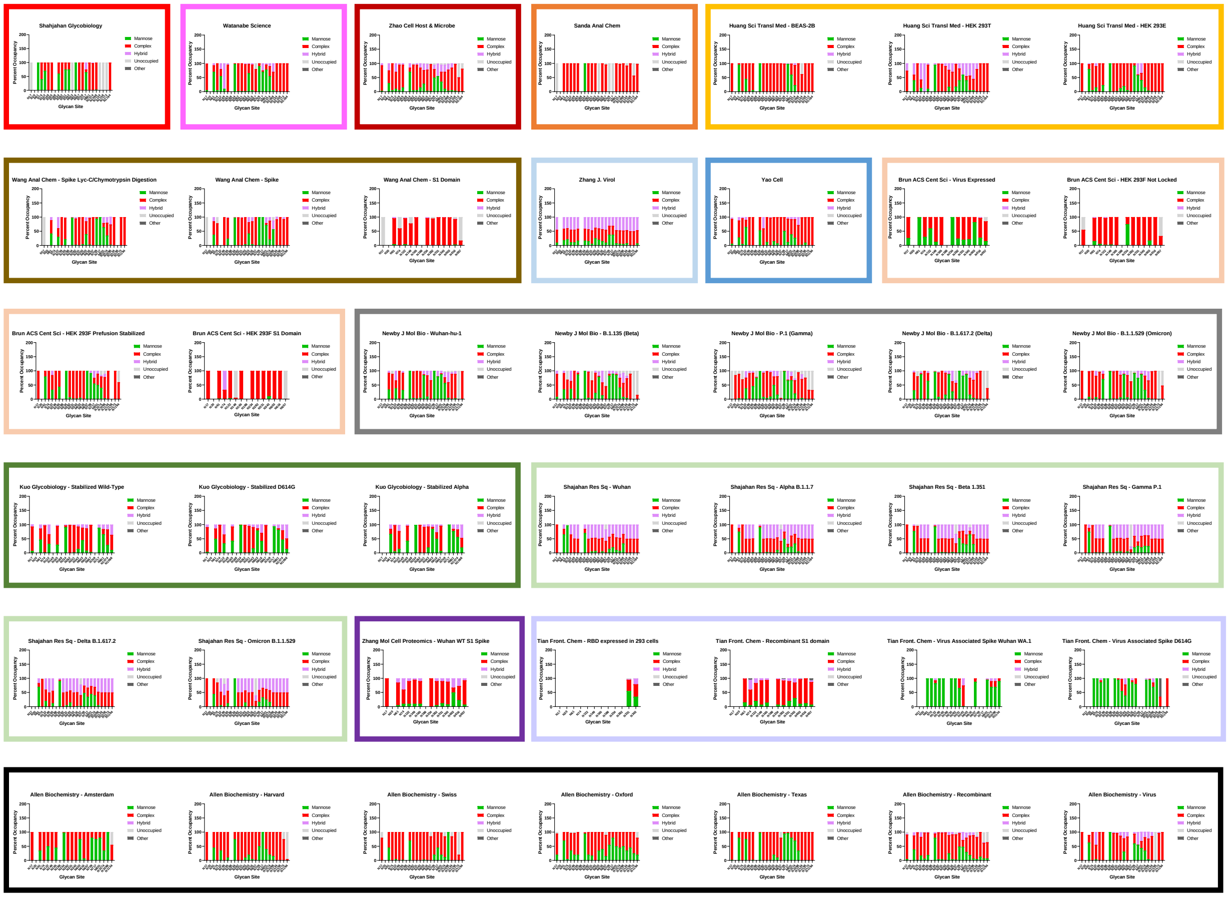

Figure S4. The glycan occupancy of the SARS-CoV-2 spike that was compiled to make Supplemental Figure 3. These data were published in the following publications listed in our references [34-37,66,103,104,108,125-131]. For publications that did not explicitly list the glycan occupancy at each site, a best estimate was given based upon their published figures.

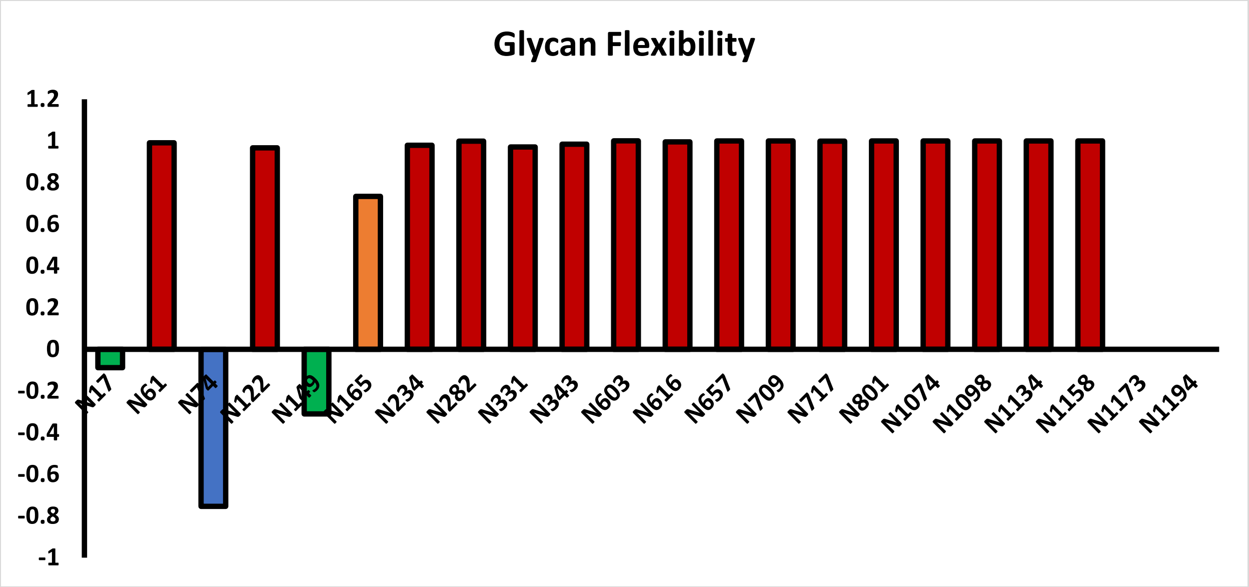

Figure S5. Analysis of SARS-CoV-2 Spike flexibility at N-linked glycan residues based on PDB structures as of July 5, 2023. To quantitate the flexibility of each glycan on the SARS-CoV-2 spike, we performed a rough analysis of all full-length SARS-CoV-2 spike cryo-EM structures in the PDB. Since the process of performing Cryo-EM microscopy entails snap-freezing samples and finding concordance between numerous "snapshots" of the protein, areas of high flexibility tend to have low resolution. Thus, we used the resolution of structures on the Protein Data Bank (PDB) as proxies for the degree of flexibility for regions of the SARS-CoV-2 spike. If each glycan residue was on a region that was resolved in the structure, we assigned it a value of 1. If the residue was not resolved, but was next to a well-resolved region, then it was assigned a value of 0. If the residue was not resolved and was not next to a well-resolved region, then it as assigned a value of -1. We averaged all these values for each residue site and then graphed them, above. Therefore, flexible regions are assigned values closer to -1 while rigid regions are assigned a value closer to 1. Bars are color coded such that consistently inflexible regions are colored dark red. Moderately inflexible regions are colored in orange. Moderately flexible regions are colored green. Consistently flexible regions are colored blue.
All spike structures that were analyzed were truncated slightly at the C-terminus, which is why there is no data for residues N1173 and N1194.

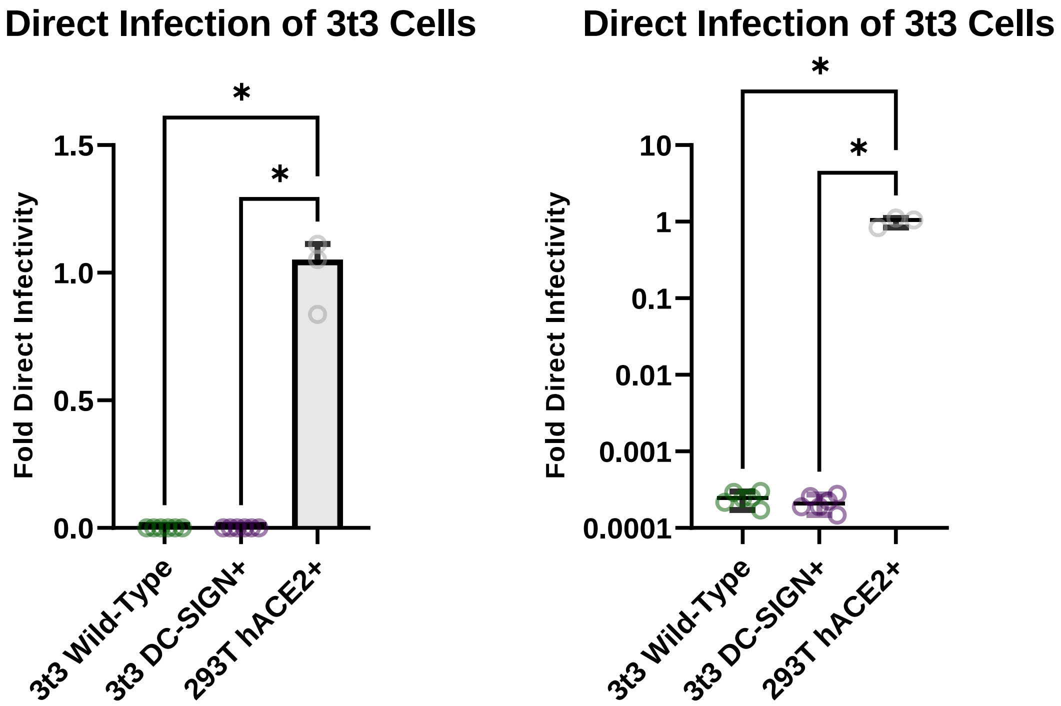

Figure S6. Verification that murine fibroblast 3t3 Wild-Type and 3t3 DC-SIGN+ cell lines are not permissible to SARS-CoV-2 infection. Briefly, 10000-15000 3t3 Wild-Type and 3t3 DC-SIGN+ cells were seeded into wells of a 96-well plate. For comparison, 10000-15000 HEK-293T hACE2+ cells were seeded into wells of a 96-well plate as well. Then, 15 μL of Wild-Type SARS-CoV-2 pseudovirus was added to wells. Infectivity assay was then returned to humidified incubator and the rest of the assay was completed as described in section 2.4. Dara shows that there is no significant difference in SARS-CoV-2 pseudovirus permissibility between 3t3 Wild-Type cells and 3t3 DC-SIGN+ cells. Both 3t3 cell lines displayed over 3 orders of magnitude less signal than SARS-CoV-2 permissive HEK-293T hACE2+ cells, indicating that 3t3 cells are a suitable cell line for virion capture for Trans-assays. Graphs depict median relative infectivity per cell type with 95% confidence interval. Statistical analysis is a Welch ANOVA with an Alpha value of 0.05. * indicates a p-value < 0.1.

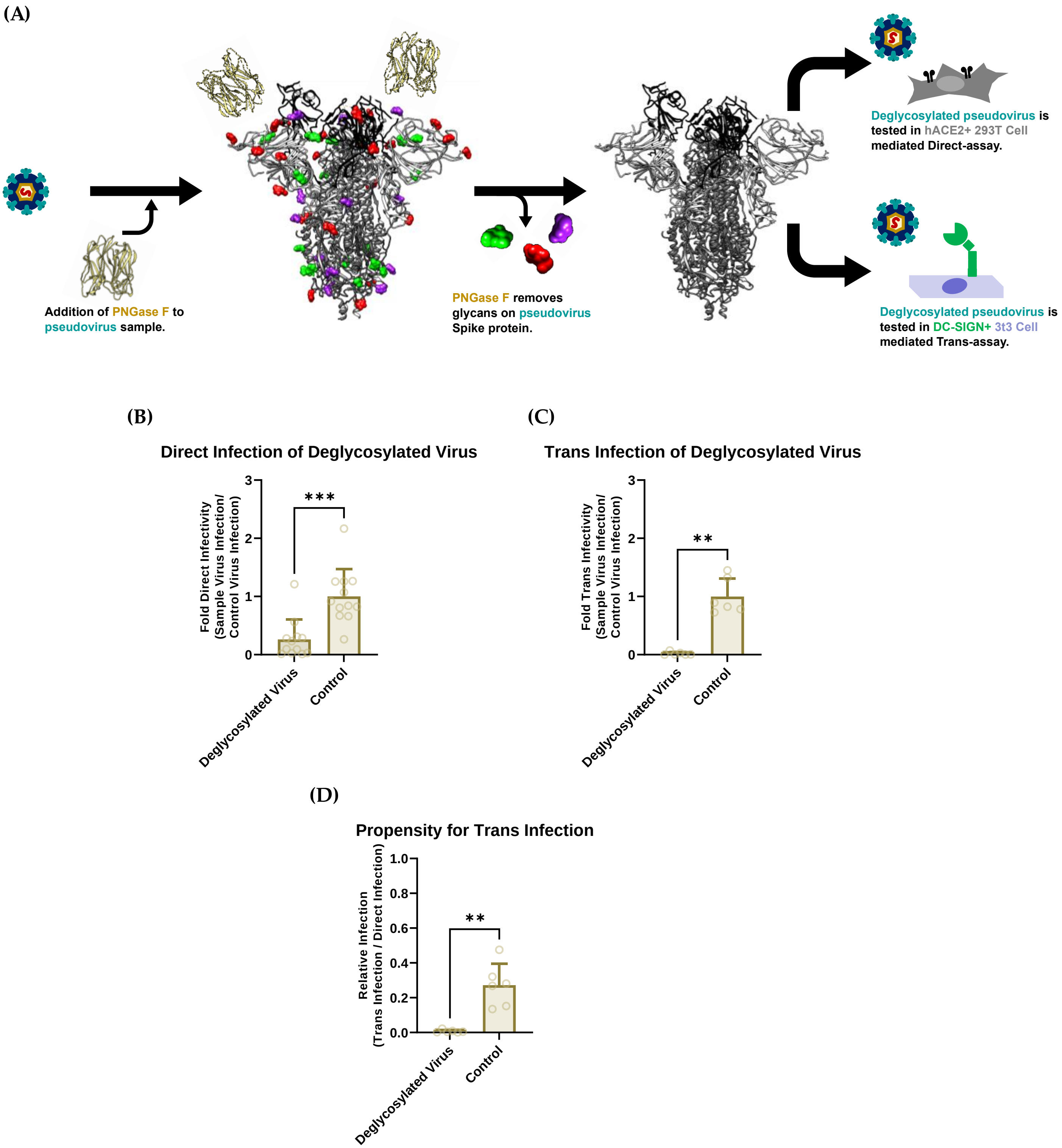

Figure S7. Deglycosylating pseudoviral samples decreases both Direct- and Trans-Infectivity. (A) Schematic diagram of our experimental procedure for PNGase F-mediated deglycosylation of Spike protein on the surface of pseudovirus. **(B)** PNGase F-mediated deglycosylation of SARS-CoV-2 pseudovirus indicates that glycosylation is important in ensuring proper function of SARS-CoV-2 Spike entry into cells via hACE2. This is expected,

based off of our results from Figure 2C. **(C)** Trans-Infectivity of SARS-CoV-2 Spike pseudovirus when exposed to PNGase F versus non-deglycosylated control. **(D)** To obtain the Propensity for Trans-Infection, the Trans-Infectivity reads for Deglycosylated and Control virus were normalized to their respective Direct-Infectivity. As expected, results appear to indicate that virus surface glycans are important in allowing for DC-SIGN-mediated recognition and Trans-infection of SARS-CoV-2 pseudotyped lentivirions. Graphs depict median relative infectivity per strain with 95% confidence interval. Statistical analysis is a Mann-Whitney T-Test. ** indicates a p-value < 0.01. *** indicates a p-value < 0.001.

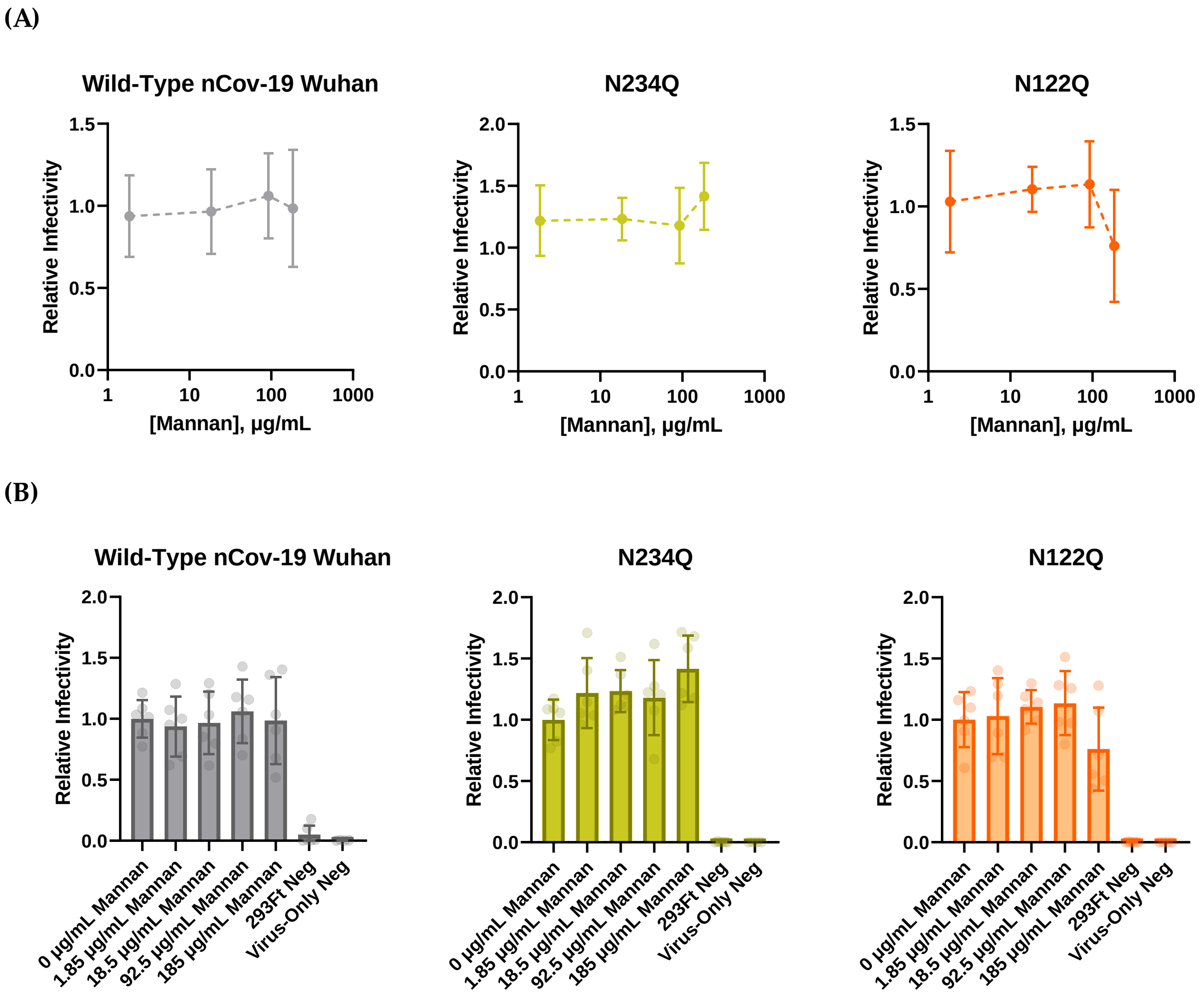

Figure S8. To ensure that Mannan had no effect on our SARS-CoV-2 pseudoviral assays beyond DC-SIGN mediated trans-infection, we performed Direct-infectivity assays with three difference strains of pseudovirus in the presence of varying concentrations of mannan. **(A)** Line graphs where each strain's mannan sample infectivity was normalized to the signal from a sample with no mannan. **(B)** Bar graphs showing the same data in addition to the controls. Overall, we found that Mannan had no significant effect on pseudovirus infectivity between 5 to 100 μg/mL. Hence, we opted to use a concentration of 20 μg/mL of mannan to inhibit DC-SIGN infection.

(A)

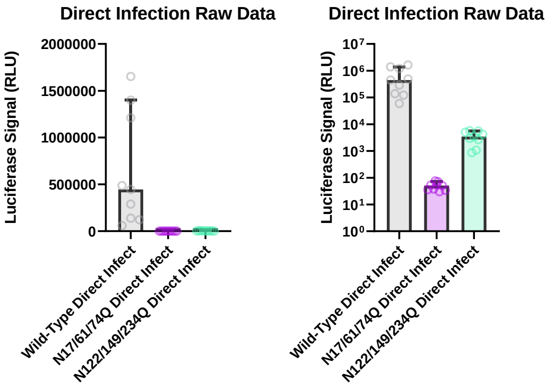

Direct Infection Raw Data
Luciferase Signal (RLU)
2000000
1500000
1000000
500000
0
Wild-Type Direct Infect
N17/61/74Q Direct Infect
N122/149/234Q Direct Infect
Direct Infection Raw Data
Luciferase Signal (RLU)
10^7
10^6
10^5
10^4
10^3
10^2
10^1
10^0
Wild-Type Direct Infect
N17/61/74Q Direct Infect
N122/149/234Q Direct Infect

(B)

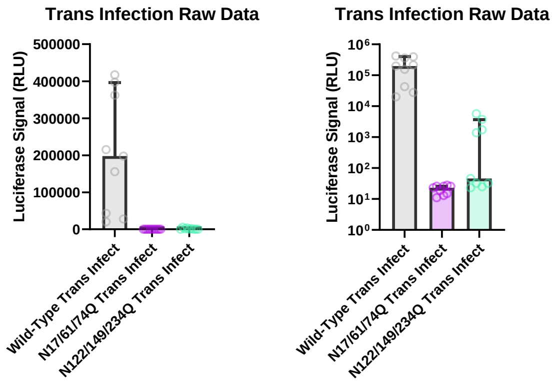

Trans Infection Raw Data
Luciferase Signal (RLU)
500000
400000
300000
200000
100000
0
Wild-Type Trans Infect
N17/61/74Q Trans Infect
N122/149/234Q Trans Infect
Trans Infection Raw Data
Luciferase Signal (RLU)
10^6
10^5
10^4
10^3
10^2
10^1
10^0
Wild-Type Trans Infect
N17/61/74Q Trans Infect
N122/149/234Q Trans Infect

(C)

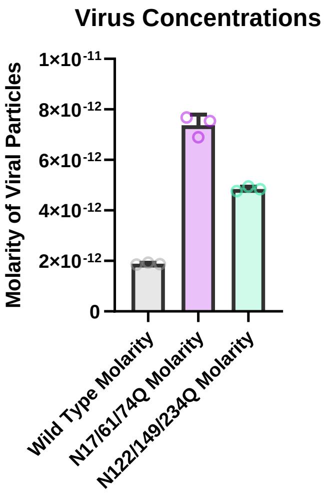

Virus Concentrations
Molarity of Viral Particles
1×10^{-11}
8×10^{-12}
6×10^{-12}
4×10^{-12}
2×10^{-12}
0
Wild Type Molarity
N17/61/74Q Molarity
N122/149/234Q Molarity

(D)

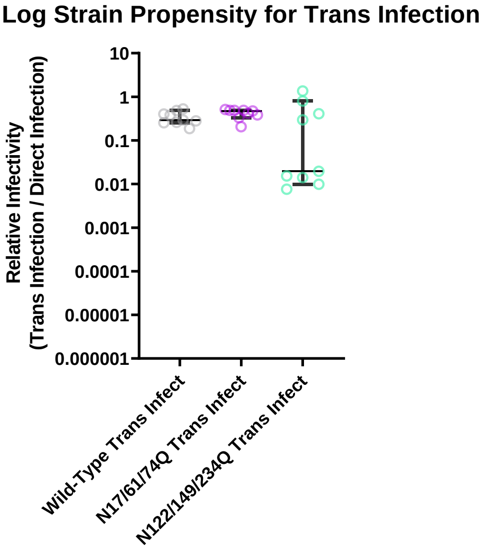

Log Strain Propensity for Trans Infection
Relative Infectivity
(Trans Infection / Direct Infection)
10
1
0.1
0.01
0.001
0.0001
0.00001
0.000001
Wild-Type Trans Infect
N17/61/74Q Trans Infect
N122/149/234Q Trans Infect

Figure S9. Cluster mutant strains show significantly lower raw infectivity signal (RLUs) than Wild-Type nCov-19 Wuhan pseudotyped virus despite having higher titers: (A) Raw Direct-infection signals for Wild-Type nCov-19 Wuhan and cluster strain pseudotyped lentivirions. (**B)** Raw Trans-infection signals for Wild-Type nCov-19 Wuhan and cluster strain pseudotyped lentivirions. Note that our instrumentation cannot reliably read below 50 RLUs, meaning that the infectivity signals for N17/61/74Q virus are too low to conclude that there is any infection compared to both cluster mutant strains. **(C)** The virus cluster mutant strains were quantified via p24 ELISA assay; the two cluster strains show greater levels of virus than the Wild-type strain, indicating that the lower signal from the cluster mutant strains is not due to low titer. Overall, even though the Wild-Type nCov-19 Wuhan pseudotyped lentivirus had less than half the molarity of either cluster strain (N17/61/74Q and N122/149/234Q), the raw luciferase reads (in relative light units, or RLU) for both Direct Infection assays and Trans Infection assays displayed at least two orders of magnitude higher RLU than either of the mutants. **(D)** Due to the low Direct- and Trans- infectivity per Molar for both cluster mutant samples, the depicted Propensity for Trans Infection samples are not an accurate reflection of the cluster mutant strains' ability to be recognized by DC-SIGN. Infectivity was essentially zero for most cluster mutant samples (the background of luminometer gives nonzero reads).

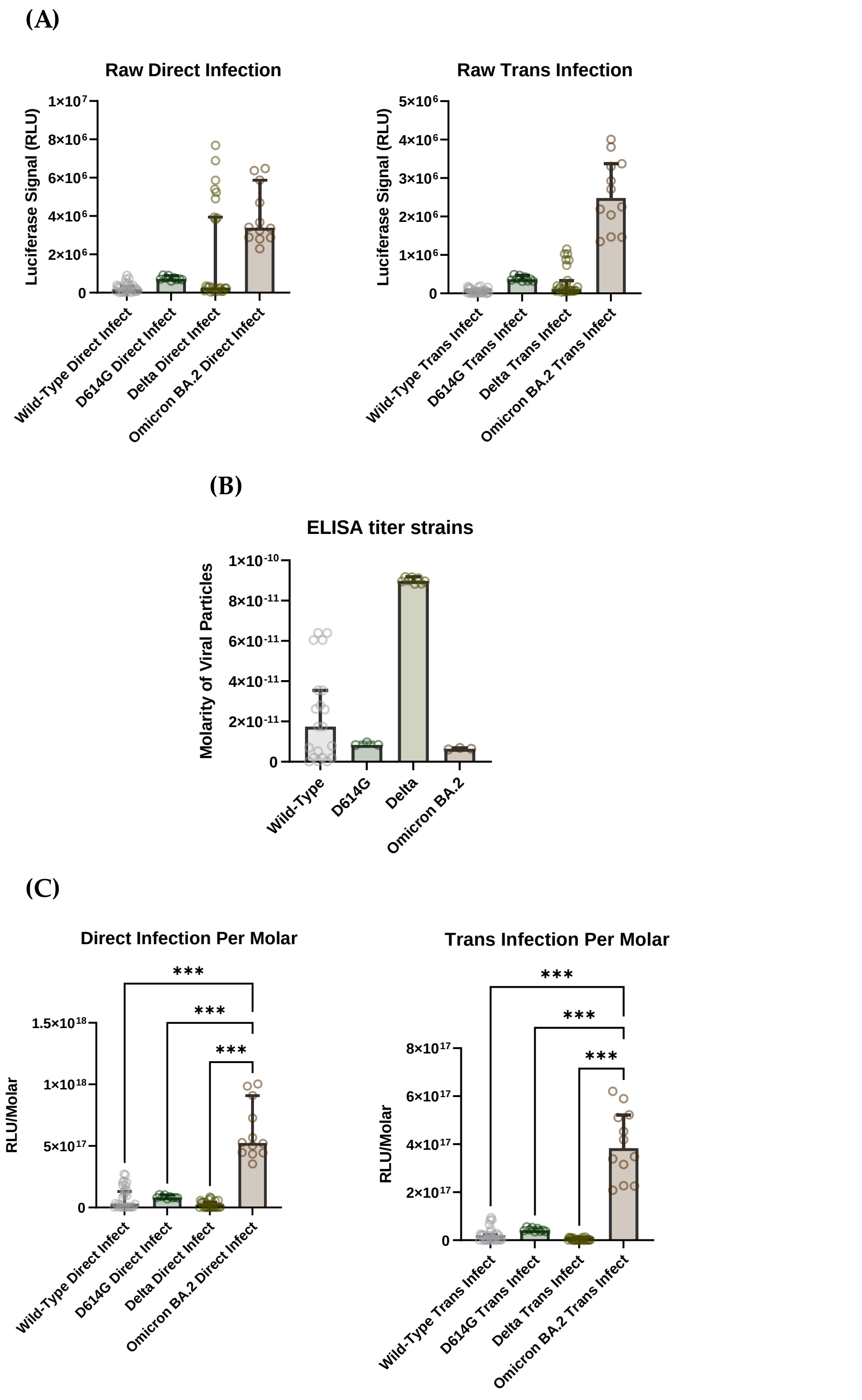

Figure S10. Demonstration of how Omicron BA.2 had the greatest hACE2-mediated infectivity on *in vitro* assays: (A). Raw infectivity values for all spike pseudotyped lentivirus. Our titer assays indicate that Delta and Omicron BA.2 strains have similar signal on Direct-infectivity assays. (**B).** Each lentiviral pseudovirus strain was titered. Even though both Delta and Omicron BA.2 strains had similar infectivity, our Delta strain virus had approximately 10 times more virus detected in the virus sample. (**C).** Infectivity for

each viral strain was normalized to the strain's molarity from the titration assay. The Omicron BA.2 lentivirus pseudovirus strain appeared to have higher infectious capability on our *in vitro* assay than the other strains. Graphs depict median relative infectivity per cell type with 95% confidence interval. Statistical analysis is a Welch ANOVA with an Alpha value of 0.05. *** indicates a p-value < 0.001.
